# Use of Antimicrobials by Class in Pigs in Germany—A Longitudinal Description Considering Different International Categorisation Systems

**DOI:** 10.3390/antibiotics11121833

**Published:** 2022-12-16

**Authors:** Clarissa Bonzelett, Anne Schnepf, Maria Hartmann, Annemarie Käsbohrer, Lothar Kreienbrock

**Affiliations:** 1 Department of Biometry, Epidemiology and Information Processing, WHO Collaborating Centre for Research and Training for Health in the Human-Animal-Environment Interface, University of Veterinary Medicine, 30559 Hannover, Germany; 2 Federal Institute for Risk Assessment, 10609 Berlin, Germany; 3 Unit for Veterinary Public Health and Epidemiology, Veterinary University Vienna, 1210 Vienna, Austria

**Keywords:** antimicrobial usage, treatment frequency, pigs, HPCIA, VCIA, Category B (restrict)

## Abstract

Antimicrobial usage in both human and veterinary medicine is considered one of the main drivers of antimicrobial resistance; its reduction poses a serious challenge. To analyse the associations between usage and resistance, data from monitoring systems and classification of all antimicrobial substances are crucial. In this analysis, we investigated longitudinal data collected between 2013 and 2020 within the Veterinary Consumption of Antibiotics project from pig farms in Germany, including all antimicrobial classes, but focusing on critically important antimicrobials: third- and fourth-generation cephalosporins, fluoroquinolones, macrolides, and polymyxins. Analysing the treatment frequency, we found that a reduction in antimicrobial use in all types of pig production has occurred over time, accompanied by a rising percentage of farms without any usage. The lists of the World Health Organisation, World Organisation for Animal Health, and European Medicine Agency classify different antimicrobial substances as critically important. The vast differences between the respective weighted treatment frequencies allocated to the antimicrobials of main interest reflect the huge impact of the three categorisation systems. We concluded that, with the aim of creating national treatment guidelines supporting veterinarians to make treatment decisions, the list of the European Medicine Agency is the most suitable.

## 1. Introduction

Antimicrobial resistance (AMR) presents an important risk for both human and animal health [1,2]. One of the major drivers of the spread of resistant bacteria is the usage of antimicrobials in all sectors [3,4]. To ensure the therapeutic efficacy in human and veterinary medicine and avoid further resistance, a need for reduction strategies through prudent use on antimicrobials became apparent [5].

International organisations from different sectors work together to address AMR. While the World Health Organisation (WHO) has provided fundamental standards—regarding the development of guidelines on prudent antimicrobial usage (AMU) in humans [6]—international organisations such as the World Organisation for Animal Health (WOAH, formerly OIE) and the European Medicine Agency (EMA), as well as national institutions, have provided such guidelines for the veterinary sector [7,8,9]. In Germany, the Federal Veterinary Chamber published such guidelines for the first time in 2000 [10].

To provide efficient strategies to manage AMR, we must gather and comprehend data on AMU in animals. For this reason, different organisations have established guidelines for monitoring programs or data collection [11,12]. However, reliable data collection on drug application in veterinary medicine has proved to be challenging. As a European activity, the European Surveillance of Veterinary Antimicrobial Consumption (ESVAC) by EMA started its monitoring in 2005 based on voluntary participation and will be mandatory by Regulation (EU) 2019/6 on veterinary medicinal products (VMPs) from 28 January 2024 onwards [13]. Published annually, the ESVAC reports provide sales data on antimicrobial consumption in animals of participating member states, with its latest report from November 2021 presenting data from 31 countries for 2019 and 2020, covering almost 100% of the food-producing animals in the EU [14]. To date, the data provided only include information on food-producing animals in general, i.e., by sales on VMPs intended for use in livestock and aggregated biomass of livestock, but without further division by species or detailed livestock classes such as dairy or fattening. Plans and guidelines are in place for collecting usage data at the species level, but they have not yet been implemented in most member states [15,16].

In addition to this European monitoring system based on sales data from pharmaceutical companies, many countries have additional national systems in place. Most of the existing national monitoring programs use sales data only, for example, reported by pharmacies and feed-mills, whereas countries such as Denmark [17] and the Netherlands [18] (monitoring started in 1994 and 2009, respectively) also use aggregated prescription data for individual farms reported by the veterinarians for their evaluations. Even so, the calculations of indicators based on prescription data are most often based on assumed standard weight and dose for animals, which may increase the uncertainty of the results [19,20].

To combat AMR, Germany’s federal government mapped the German Antibiotic Resistance Strategy (“Deutsche Antibiotika Resistenz Strategie” (DART)) in 2008, which aims to control the spread of resistance [21]. Following the need for monitoring programs, different national systems for data collection have been established. The official collection of VMPS sales data was started in 2011 by the German Institute for Medicinal Documentation and Information (“Deutsches Institut für Medizinische Dokumentation und Information” (DIMDI)) and from January 2022 onwards by the jurisdiction of the Federal Office of Consumer Protection and Food Safety (“Bundesamt für Verbraucherschutz und Lebensmittelsicherheit” (BVL)) [22,23,24].

To collect data on AMU and implement a benchmarking system, the German Medicinal Products Act (“Arzneimittelgesetz”, and from 28 January 2022 onwards, by the Veterinary Medicinal Products Act, “Tierarzneimittelgesetz”) underwent its 16th amendment in 2014. From then on, data collection on antimicrobial use to calculate a treatment frequency (TF) per half year for broilers, turkeys, veal calves, beef cattle, weaners, and fattening pigs became mandatory for all farms larger than a defined size [25]. In addition, the scientific project Veterinary Consumption of Antibiotics (VetCAb) has enrolled AMU monitoring data in German livestock husbandry at the species level since 2007 in a sentinel study with voluntary participation [26].

In 2003 and 2004, expert workshops (“Non-Human Antimicrobial Usage and Antimicrobial Resistance”) by the Food and Agriculture Organisation of the United Nations, WOAH, and WHO took place on resistance to antimicrobials representing a “global public and animal health concern”, and that it is impacted by AMU in all sectors [27]. As a result, the participating organisations decided that the WHO would develop a list of critically important antimicrobial substances for human medicine [28], while the WOAH would do so for substances important for animal health and welfare [29]. In a third activity, the EMA created a list in 2014, following a request by the European Commission, combining both existing lists, resulting in a more balanced view between animal and public health. This list focuses on the need to maintain individual antimicrobial classes for human health and the risk of AMR transfer from animals to humans, as well as on the availability of antimicrobials with lower resistance potential as alternative treatment choices in veterinary medicine [30].

Therefore, in this study, we aimed to assess the practicability of the existing lists as the basis for national treatment guidelines, including classification into first-, second-, and third-line antimicrobials. To this end, this manuscript presents results for the individual antimicrobial substances used to treat pigs in the VetCAb project by using the TF and weighted treatment frequency (TF_w%_), as well as the results grouped by list classification. In addition, we evaluated typical indications for treatment.

## 2. Results

### 2.1. Description of Study Population

In total, we collected 141,190 application and delivery forms (ADFs) for pigs over the study period: 32,534 for piglets, 38,781 for sows, 24,585 for weaners, and the most for fattening pigs, with a total of 45,290 prescriptions. The number of holdings of piglets fluctuated between 139 and 250, with 1363–2969 ADFs (median: approximately 11 ADFs per holding per half year); for sows, it fluctuated between 139 and 256 holdings, with 1814–3287 ADFs (median: approximately 13 ADFs per holding per half year). For weaners, it fluctuated between 156 and 305 holdings, with 1037–2549 ADFs (median: approximately 6 ADFs per holding per half year); for fattening pigs, it fluctuated between 413 and 768, with 1934–4326 ADFs (median: approximately 5 ADFs per holding per half year).

Participating veterinarians and their respective farms are mostly located in middle and northwest Germany. As described by van Rennings et al. [31], we divided farms into small, medium, and large, based on the number of livestock places (number of kept animals) per farm, with different cut-offs for the four production types. Weaners (from 330 animals up), fattening pigs (370 up), and sows (mostly 200 animals and more) were mainly kept on farms classified as large, whereas piglets were equally distributed among all farm sizes.

### 2.2. Total Treatment with Antimicrobials

During the study period, the percentage of holdings without antimicrobial usage increased in all four types of production, with some fluctuations between the half years. The largest change occurred in piglets, where the percentage of holdings without any AMU rose from 8.3% in 2013-1 to 39.5% in 2020-2. For the other production types, these changes were between 15.2% and 34.6% for sows, 24.4% and 42.1% for weaners, and 22.8% and 39.2% for fattening pigs.

**Figure 1:**
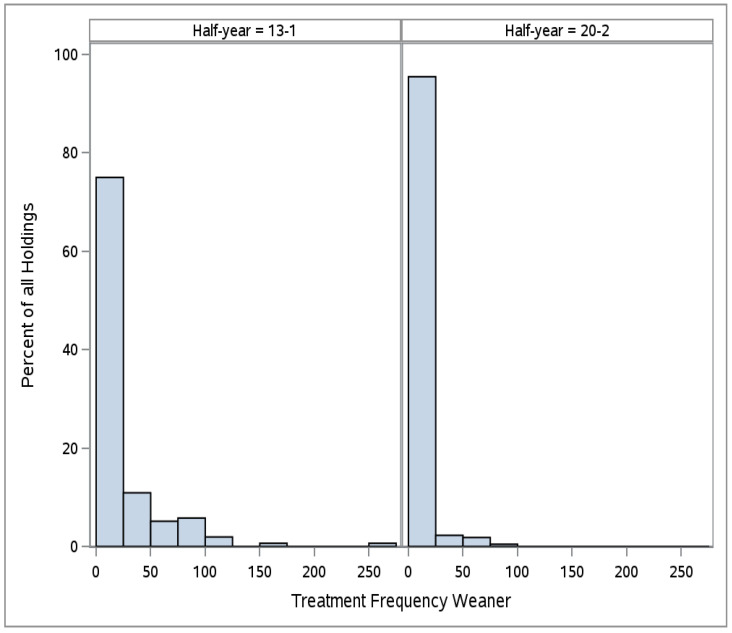
Distribution of treatment frequencies of weaners in 2013-1 (13-1) and 2020-2 (20-2).

Concerning the total treatment frequency for all production types in all half years, we observed a right-skewed distribution over the collective of farms (see Figure 1 as an example). The median decreased in all groups, i.e., from 4.0 to 0.8 (−80%) in piglets, 1.0 to 0.6 (−40%) in sows, 7.4 to 0.3 (−96%) in weaners, and from 2.5 to 0.1 (−96%) in fattening pigs (see Table S1).

Regarding the weighted treatment frequency (TF_w%_; Formula (4)), the main indications for AMU in our study differed between the four groups. They were (range given over study period), for piglets, mostly respiratory (20–50%) and joint (5–50%), followed by intestinal diseases (5–40%); for sows, mostly respiratory (20–70%), urogenital (10–25%), and “other” diseases (10–50%, including infections caused by bacteria such as *Streptococci*); and for weaners and fattening pigs, respiratory (50–70%) and intestinal diseases (20–40%). For more information on this topic, see Tables S2–S5.

### 2.3. Treatment Frequency by Antimicrobial Class

The antimicrobial substances used in veterinary medicine belong to 12 different classes (Aminoglycosides, Amphenicoles, Cephalosporins, Fluoroquinolones, Lincosamides, Macrolides, Penicillins, Pleuromutilins, Polymyxins, Sulfonamides, Tetracyclines and Trimethoprim). With a focus on the critical antimicrobials, we analysed the TF_w%_ for each production type divided by list classification, antimicrobial class, and active substance. The distributional patterns of use varied considerably.

#### 2.3.1. Piglets

Antimicrobial classes with the highest weighted treatment frequency in this production type were penicillins, macrolides, and aminoglycosides.

The TF_w%_ for HPCIAs fluctuated throughout the study period, which mostly related to the usage of macrolides (tulathromycin, ≤34.8%) and colistin (≤16.4%). Additionally, third- and fourth-generation cephalosporins, for which the TF_w%_ was highest in this group (≤7.4%), and fluoroquinolones also affect the HPCIA percentages.

In contrast, the TF_w%_ for VCIAs was mainly influenced by polymyxins’ usage. For the first few half years, colistin percentages reached 16.4%, resulting in a relatively low starting point of the TF_w%_ for VCIAs, before dropping to <1% in 2016, followed by another rise starting in 2019.

The TF_w%_ for Category B fluctuated throughout the study period, mainly correlated with the usage of fluoroquinolones (enrofloxacin) and colistin.

For more information on this topic, see Table 1, Table S6 and Figure S1.

#### 2.3.2. Sows

Antimicrobial classes with the highest weighted treatment frequency in this production type were penicillins, tetracyclines, sulfonamides, and trimethoprim.

The TF_w%_ for HPCIAs was high in 2013-1 due to a high usage of macrolides (tylosin), before it dropped in the following half year (−31.2%). Afterwards, the values fluctuated. The TF_w%_ for fluoroquinolones (mainly enrofloxacin) was highest in this group, reaching 11.4%, whereas the TF_w%_ for third- and fourth-generation cephalosporins varied (cefquinome).

The TF_w%_ for VCIAs was even higher, mostly > 95%. In this production type, the uses of colistin (<3%), lincomycin, and tiamulin were low, with only a higher percentage of colistin in 2014 resulting in a slight reduction in the VCIAs.

The TF_w%_ for Category B fluctuated for the first three years of our study due to varying percentages of fluoroquinolones and polymyxins. Starting in 2017-1, the percentage dropped, reaching a minimum in 2018-2 (2.7%), around which most values remained.

For more information on this topic, see Table 2, Table S7 and Figure S2.

#### 2.3.3. Weaners

Antimicrobial classes with the highest weighted treatment frequency in this production type were penicillins, polymyxins, and tetracyclines.

The TF_w%_ for HPCIAs remained relatively stable, with an overall decreasing trend. Fluctuations closely related to the usage of polymyxins, because they accounted for the largest part of the HPCIAs in this group (overall reduction of −19.2%). In addition, macrolides (mainly tulathromycin and tylosin) influenced the HPCIAs TF_w%_, whereas the percentages for third- and fourth-generation cephalosporins as well as fluoroquinolones remained low (≤1%).

The TF_w%_ for VCIAs was lowest in this group, with a minimum of 63.0%, which strongly related to the relatively high usage of colistin (≤36.1%).

The TF_w%_ for Category B was highest in this group: For the first two years, the values stayed >30%. Starting in 2015, the percentage dropped (minimum in 2016-2), followed by ongoing fluctuation, mostly related to polymyxins usage.

For more information on this topic, see Table 3, Table S8 and Figure S3.

#### 2.3.4. Fattening Pigs

The antimicrobial classes with the highest weighted treatment frequency in this production type were penicillins, tetracyclines, and macrolides.

The TF_w%_ for HPCIAs varied, mainly due to the uses of macrolides (tylosin, ≤23.2%) and colistin (overall decreasing trend). The percentages for third- and fourth-generation cephalosporins and for fluoroquinolones remained low (<3.2%).

The TF_w%_ for VCIAs fluctuated, with a minimum of 75.9%, due to tiamulin (≤15.8%), colistin, and lincosamin usage.

The TF_w%_ for Category B was lowest in this group, related to the relative low usages of cephalosporins, fluoroquinolones, and polymyxins. The values were mainly <10%, excluding 2013-2 to 2014-2, when colistin usage rose.

For more information on this topic, see Table 4, Table S9 and Figure S4.

## 3. Discussion

In this study, we obtained longitudinal data on the AMU for pigs from Germany between the years 2013 and 2020 by measuring the treatment frequencies as described for the antimicrobial classes and substances and different indications for treatment. In addition, we analysed the usage patterns in the form of weighted TF by different classification systems, concentrating on the antimicrobials considered the highest priority critically important: third- and fourth-generation cephalosporins, fluoroquinolones, polymyxins, and macrolides. In general, the results of this analysis should help with assessing the practicability of the existing lists so that these lists can be implemented into national treatment guidelines, including dividing the list into first-, second-, and third-line antimicrobials. Such guidelines should support veterinarians in making treatment decisions.

### 3.1. Evaluation of Methods and Results

The TF methods of analyses presented here (Formula (2)) follow the concept reported by Merle et al. [26]. The TF is consistent with the therapy incidence (TI) used by other authors [32,33,34]. However, unlike most calculations, we used the current body weight under therapy and the used daily doses (UDDs) in place of an average body weight and defined daily dose animal (DDD; the assumed average dose per kilogram of animal per species per day) [35]. In general, these calculations are more precise, as the ADFs in Germany contain the necessary data on the total amount of antimicrobials dispensed by number of animals treated and treatment days, enabling the direct use of the UDD. Following the results from Kasabova et al. [20]—where the median TF_UDD_ for piglets was 3.4 and the TF_DDD_ was 6.2, and the median TF_UDD_ for fattening pigs was 4.7 and the TF_DDD_ was 5.6—we regarded the UDD as being more accurate and less prone to bias. In conclusion, in production types, where the weights of the animals vary considerably, the DDD underestimates the TF of animals weighing less than the standard weight at the beginning of a production cycle, whereas it overestimates the TF at the end of a cycle when the animals weigh more than the standard weight.

Because the ADFs also indicate the production type of a treated animal species, we separately analysed the AMU for the different groups, the necessity of which has been described by authors such as Jensen et al. [36] and the EMA [14,15,16]. Similarly, in the German official system, separate benchmarking values are calculated for weaners and fattening pigs. In our study, we stratified pig production into four groups based on the information given in the ADFs: sows, piglets, weaners, and fattening pigs [31]. Other authors summarised sows and piglets [36,37] or analysed data for all production types combined [38], which may have restricted the direct comparability of their results with those of other studies [39,40].

### 3.2. Data Quality

Because our study was based on voluntary participation, selection bias and migration bias could not be completely ruled out, meaning that the actual AMU might have been higher or lower than reported in our data. However, the TF range results reported by Rennings et al. [31] suggested that both biases are most likely low. Furthermore, the overall reduction trends of the TF from our study are similar to the trends of the official benchmarking system, as described in the following chapter “AMU changes”. Therefore, we conclude, that our data is representative for the German pig population.

Another bias might have occurred due to ADF misallocation: In general, using the ADFs allows differentiating between the production types, and therefore increases the accuracy of an analysis of usage data for the individual production types compared with that of calculations using standardised weight and DDDs. Misclassification may have appeared if the treatment for one production type was documented for another or vice versa. In addition, errors were possible due to the transfer of handwritten records into the database, as shown by Hartel et al. and others [41,42]. Because the misallocation applies not only to our data but also to the official data, we concluded that the use of our data is acceptable.

### 3.3. AMU Changes

In our study, an overall reduction occurred in the total treatment frequency in all types of production, which was accompanied by a rise in the number of holdings without any antimicrobial usage. This is in agreement with the new legislation issued in 2013 demanding an overall reduction in AMU [25]. It also applies to the TF documented by the official benchmarking system, where the TF for weaners dropped from 4.793 to 2.759 (−42%) and for fattening pigs from 1.199 to 0.417 (−65%) between 2015 and 2020 [43]. The reductions in the TF in our study for the same period are higher than reported in the official numbers: weaners (−90%), fattening pigs (−50%), piglets (−67%), and sows (−33%). This could indicate that participating veterinarians were more interested in reducing AMU. However, as the general reducing trends were the same, we assume that this did not have a substantial impact on the overall pattern of reduction.

The overall reduction also applied to the amount of antimicrobial substances sold in Germany for all animal species, as documented by the official reports on sales data. Here, a reduction of −60% occurred between 2011 and 2019 [44]. For 2020, the official results show a rise in sales data on antimicrobial substances for the first time since 2011 (+4.6%), as well as a rise in the official TF (+2.7% for weaners and +16.6% for fattening pigs) [43], but not in the TF in our data.

The rise in sales data also applied to many other European countries, with an overall increase of +5.8%, according to the ESVAC report [14]. For 2021, sales data in Germany decreased by −14% [45], while the official TF decreased (−33% for weaners and −28.7% for fattening pigs). This showed that the rise in 2020 was only temporary—possibly caused by an excessive purchase of VMPs in 2020 due to the COVID-19 pandemic and the Brexit or data anomaly, as assumed in the ESVAC report [14]—and that sales and TF continued to decrease thereafter.

In addition to this overall reduction, the pattern of drug classes applied changed, resulting in changes in the weighted treatment frequency of some antimicrobial classes and substances by production type, indicating that the habits of drug administration changed. We discuss these changes below for the individual classes.

When examining the changes in the TF_w%_ of an antimicrobial class, the total reduction in one antimicrobial class is the product of multiplying the TF_w%_ by the total TF and then comparing the half years. For simplicity, we chose to describe only the TF_w%_ in this manuscript.

#### 3.3.1. Cephalosporins and Fluoroquinolones

Our results showed that during the course of our study, the roles of cephalosporins and fluoroquinolones in all production types remained around the same level.

The TF_w%_ remained at ≤0.5% for cephalosporins and ≤3.1% for fluoroquinolones in both weaners and fattening pigs. Slightly higher percentages for fluoroquinolones in fattening pigs were related to the treatment of respiratory and intestinal diseases with this antimicrobial class. From those findings, we concluded that, in our study, these two antimicrobial classes were only rarely administered as third-line antimicrobials, if no alternative was available in either type of production, in accordance with findings from other authors [39,46]. This is probably also due to the preferred administration route being oral in both production types—more suited for group treatment—and both antimicrobial classes are parenterally administered [46,47,48].

In sows, the TF_w%_ for cephalosporins remained constant (≤4.3%) during the observation period, highlighting the role of this class for the treatment of mainly reproductive (postpartum dysgalactia syndrome), respiratory, and “other” diseases such as septicaemia and polyserositis. These antimicrobials are primarily administered parenterally to individual sick animals as, often, only a low number of sows are affected [49,50,51]. The TF_w%_ of fluoroquinolones remained relatively high until 2019-1, before it decreased. The indications for their use are intestinal, urogenital, and respiratory diseases [48,50,51]. The treatment frequencies of these diseases decreased in our study (see Table S3), which could indicate that the occurrences of these diseases also decreased.

In piglets, cephalosporin usage fluctuated, but stayed mostly at <5%. They are preferred due to their long and potent effect and low dosages [48,52]. Their typical indications are respiratory and joint diseases caused by bacteria such as *Streptococcus suis*, as well as “other” diseases such as after birth, castration, and teeth clipping, which is in accordance with findings reported by other authors [32,39,53,54].

The usage of fluoroquinolones in piglets also fluctuated, and they were mostly administered for intestinal diseases, which, according to the participating veterinarians, were mainly caused by enrofloxacin-susceptible *E. coli*. This is in accordance with the findings of other authors [32,55].

Both cephalosporins and fluoroquinolones are administered parenterally to piglets—as most available products are only licenced for parenteral use—indicating individual treatments of the animals.

Compared with other substance classes, these TF_w%_ are still moderately low and only account for a small proportion of the overall AMU. According to the ESVAC report, overall sales of cephalosporins decreased by −32.8%: all participating countries documented low levels of cephalosporin sales, with Germany placing in the middle (−50%). Sales of fluoroquinolones reduced overall by −12.8%—with a larger variation throughout the participating countries—with Germany placing in the lower third [14].

In our study, fluoroquinolone and cephalosporin usage reduced simultaneously with total AMU consumption, which was reflected by the constant TF_w%_. This indicates that although the use of both reduced (in line with the official reports), the reliance on these antimicrobial classes remained. In contrast, if only considering the period from 2017-2 (before the new legislation) to 2020-2, we observed a notable decline in the use of both antimicrobial classes for piglets and sows, but this trend did not continue after 2018-2. This suggests that the new legislation (mandatory antimicrobial susceptibility testing prior to the use of third- and fourth-generation cephalosporins and fluoroquinolones since March 2018 [56]) had only a time-limited effect on the use in both production types—where the usage of these antimicrobials played an important role—but was not sufficient to reduce the use of these antimicrobials in our study population in the long term. This indicates that further efforts are needed to continue this trend.

In comparison, other countries have a ban on cephalosporin and fluoroquinolone usage in pigs [17,57], at least raising the question if phasing out these classes—without risking animal health by over-limiting treatment options—is also possible in Germany. Denmark has a mandatory ban on the use of fluoroquinolones (zero usage) and a voluntary ban on the use of third- and fourth-generation cephalosporins (≤1%), while regulations in Norway reduced the limit on the administration of both classes to less than 0.01% [14]. Both countries have not reported a rise in animal deaths due to a lack of available treatment options caused by these restrictions. This highlights the need to investigate how these countries treat the infections that are treated with these two classes in Germany, and to investigate if the usage of these antimicrobial classes can also be phased out in Germany.

#### 3.3.2. Polymyxins

Our findings showed a reduction in the TF_w%_ for polymyxins in all four types of production: the higher TF_w%_ in sows in 2014 was related to the higher usage of colistin for the treatment of intestinal diseases. From the original data, whether this was due to the treatment of sows (probably preventive measures) and piglets together as a unit or to the incorrect assignment of ADF from piglets to sows by farmers is unclear. However, the TF_w%_ for colistin decreased drastically in 2015 and remained low. The rare use of colistin in sows is also supported by other researchers’ findings [31,46,53]. The typical indications for treatment in sows are respiratory and urogenital diseases, lameness, and “others” (e.g., systemic infection and sepsis), for which no colistin use was reported in our study. Therefore, we concluded that colistin played a less prominent role in the treatment of sows, with the higher usage in 2014 likely connected to a misallocation of ADFs.

Weaners represent the group in our study to which veterinarians administered polymyxins most often. This was mostly related to the treatment or prevention of post-weaning diarrhoea caused by *E. coli*, a widespread disease in weaning pigs [32,36,50]. In addition, colistin is mostly administered orally, making it suitable for group treatment [32,58]. The TF_w%_ in our study dropped from 32.9% to 19.2%, in line with the decreasing trend in polymyxin sales from the official reports for 2020 compared with 2013 (−51.8%) [44]. The reduction could have been caused by different means such as improving management and biosecurity [53,59,60], vaccination against *E. coli* [61,62,63], changing to different treatment options such as zinc oxide ([64,65]), and the monitoring system [25,66].

Compared with weaners, colistin played a less important role for the piglets and fattening pigs in our study population. In piglets, infections caused by *E. coli* (additional comments in our data were provided by the veterinarians) were also treated with fluoroquinolones, which was also described in the literature [55]. In fattening pigs, intestinal diseases are often caused by different bacteria such as *Brachyspira* or *Lawsonia intracellularis*, which are treated with different antimicrobial substances [40,46,51]. Nevertheless, in further investigations, researchers should closely monitor the indications and periods of polymyxins’ administration in these production types to analyse if an additional reduction in this antimicrobial substance is feasible.

According to the ESVAC, the overall sales of polymyxins for all food-producing animals in Europe decreased by almost −77%, while sales in Germany declined by −50% [14], which was still higher than the requested maximum of 5.0 mg/population correction unit (PCU). Thus, further reduction strategies should be implemented. In the Netherlands, polymyxin sales for pigs in general (no division by production type available) decreased until 2017, followed by an increase, in contrast with the overall reduction we noted in our study. As a result of this increase and in accordance with the HPCIA classification, the Dutch regard polymyxins as third-choice antimicrobials and aim to phase out their usage from 2021 onwards [18]. How this will affect sales and the use of the other antimicrobial classes remains to be seen. In accordance with our data, weaners were the production type most often treated with colistin in Switzerland [46,67], which was also the case in Denmark [17] before the government prohibited the use of colistin by 2017.

Zinc oxide, which is the only non-antibiotic alternative for the treatment of gastrointestinal infections caused by resistant *E. coli* in pigs otherwise treated with colistin, appears to be associated with co-selection for methicillin-resistant *Staphylococcus aureus* and environmental contamination [64,68,69]. As a result, the European Commission decided on a zinc oxide ban in the EU starting in 2022 [70], which will probably cause a rise in colistin consumption in European countries, where zinc oxide is administered more regularly than in Germany (e.g., Denmark: almost 500 tonnes in 2020 [17]). How countries—especially those where the use of colistin is also banned—react to this new legislation will need to be observed closely.

#### 3.3.3. Macrolides

In contrast with polymyxins, we observed no clear trend in macrolide usage. Regarding the findings in our study, macrolides played an important role in treating piglets—where the TF_w%_ for macrolides fluctuated between the half years up to 35.5%—and fattening pigs, whereas their role in sows (except 2013-1) and weaners was much less prominent. Macrolides were most often used for the treatment of respiratory and intestinal diseases, in accordance with results from other studies [50,51].

In piglets, the TF_w%_ for macrolides fluctuated, with an increasing trend, highlighting the role of these antimicrobials for this production type. This was especially true for the treatment of respiratory diseases—the most common disease complex in piglets in our study (as well as in other studies)—followed by intestinal and joint diseases [19,31,50,54]. Callens et al. described that tulathromycin—which was also the most frequently administered macrolide in our study—was often used in combination with iron mineral preparations at an early age to prevent coughing and sneezing as well as iron deficiency [54]. The fluctuations in the TF_w%_ resulted from shifting frequencies of administrations, pattern shifts to other antimicrobials (mostly penicillins), a rise in holdings without AMU, and the overall drop in AMU.

For fattening pigs, the TF_w%_ for macrolides remained relative constant (≤23.6%), even though the total TF decreased. These results suggested that the relevance of this antimicrobial class stayed nearly the same over the study period, even though a total reduction occurred. The indications for usage are respiratory—such as enzootic pneumonia caused by bacteria such as *Actinobacillus pleuropneumoniae*, *Pasteurella multocida*, *Mycoplasma hyopneumoniae*, *Glaesserella parasuis*, and *Bordetella bronchiseptica*—and intestinal diseases such as swine dysentery and porcine proliferative enteropathy [36,40,51,54]. Existing fluctuations were mostly related to AMU pattern shifts: mainly penicillins, tetracyclines, and pleuromutilins.

For further reduction of macrolides in pigs, biosecurity and management strategies must be increased and optimised, and their indication-usage relationship must be monitored more closely, especially in piglets and fattening pigs.

According to the ESVAC report, sales of macrolides increased in Germany in 2020 (in alignment with increased overall sales, as mentioned above), but compared with 2011, the sales still decreased. In comparison with other countries, Germany ranked somewhere in the middle, hinting that a further reduction in the use of this antimicrobial class is possible [14]. In Denmark, macrolides represent the only highest priority critically important antimicrobials still used for treating pigs. Their usage was highest in weaners, with a steady increase—especially since 2017; they were probably used for the treatment of diseases previously treated with colistin—representing the second-most administered class of antimicrobials in this production type. In fattening pigs, sows, and piglets, the level of macrolide consumption varied but, with an increasing trend since 2017, ranked in third and second places in the antimicrobial classes most administered. This could be a reaction to the ban on all other critically important antimicrobials in Denmark, highlighting the importance of this class in lieu of alternatives [17]. In contrast, macrolides were most frequently used in our study in fattening pigs (respiratory and intestinal diseases) and piglets (respiratory diseases) and played only a secondary role in weaners and sows. In the Netherlands, macrolides and lincosamides form a combined number, even further complicating direct comparison. For pigs in general, the sales of macrolides and lincosamides remained nearly the same, in contrast with the decreasing trend noted in our study [18].

#### 3.3.4. Other Antimicrobial Classes

In addition to the antimicrobial classes addressed above, classical antimicrobials—such as penicillins, tetracyclines, and aminoglycosides—played an important role in the treatment of pigs in Germany. Penicillins were the most frequently administered class in all types of production (15–60%) and were used to treat diseases from all indication groups. Tetracyclines were the second-most administered class in sows (15–40%)—for respiratory and “other” diseases—and fattening pigs (~30%)—mostly for respiratory diseases (in some years, also for skin diseases and the central nervous system)—and were the third-most administered class in weaners (~20%)—mainly for respiratory diseases. Aminoglycosides only played a role in the treatment of piglets (third administered class, 10–20%), where this class was used for the treatment of intestinal, joint, and “other” diseases. Sulfonamides and trimethoprim were the third-most commonly administered classes in sows (8–20%) and were used to treat urogenital, respiratory, intestinal, and “other” diseases.

### 3.4. Use of Classification Systems

All antimicrobial classes discussed above are covered by the various classification systems proposed by different organisations. Notably, the existing lists have different intentions for use and therefore must be treated differently. As the WHO list focuses on human health and the WOAH list on animal health, the EMA list may be quoted as an attempt to balance both views.

To categorise the different antimicrobial classes, the WHO defined two criteria: The first states that an “antimicrobial class is the sole or one of limited available therapies to treat serious bacterial infections in humans”. The second expresses that the used antimicrobial class treats infections in people caused by bacteria that may acquire resistance genes or transmit from non-human sources. In accordance, the WHO considers the global AMU and regards substances as important for humans if they have indications in human medicine anywhere in the world, regardless if the cause for these diseases is bacteria common to animals or humans [71]. Thus, this list does not consider the importance of antimicrobial substances for veterinary medicine. Although such an approach facilitates the trade and general comparison of AMU, it does not consider the specific requirements of individual countries, as it is a global approach that does not distinguish whether a particular disease requiring a particular antimicrobial substance actually occurs in a particular country or region. Antimicrobials classified as HPCIAs, which veterinarians dispense, include the third- and fourth-generation cephalosporins, quinolones, macrolides, and polymyxins. From a One Health approach, this classification must be considered for creating a list for veterinary medicine, but for the same reason, a solely human-based approach is insufficient.

The WOAH list [29] (last updated in 2019) addresses antimicrobial substances authorised for food-producing animals, does not include substances solely used in human medicine, does not include growth-promoters, and focuses mostly on antibacterial substances. Furthermore, the document advises against the prophylactic usage of antimicrobials classified as HPCIAs by the WHO; against usage of HPCIAs if other, less critical antimicrobials are available instead; and on necessary administration only after bacteriological testing for resistance.

For our data, an analysis of the AMU for the different antimicrobial substances classified by the WOAH with regard to their varying significance remains futile. Of the 36 substances used, only three—colistin, lincosamin, and tiamulin—were not VCIAs, resulting in very high TF_w%_ values for the VCIAs in all four types of production, especially with low colistin usage. This list, in its current form, is consequently not aligned with the One Health concept, because it focuses on the general need for veterinary application only. Therefore, following the global conference on AMR in 2018, the WOAH decided to create additional antimicrobial lists of veterinary importance by species [72]. Such a list, e.g., for pigs, would represent more accurate guideline on which antimicrobials are of critical importance for this animal species and would thus be a more appropriate tool for comparing AMU and its relevance regarding human and animal health.

Remarkably, colistin is only listed as highly, and not critically, important for animals in general [29], and many countries around the world use only small amounts of colistin to treat animals or have prohibited its use. This is in contrast with Germany, where it is regularly administered in veterinary medicine and where various authors have described a lack of alternatives for certain infectious diseases in animals [14,64,73]. Until recently, only veterinarians used colistin due to its toxicity in humans, making it a more appropriate choice for usage in veterinary medicine than cephalosporins and fluoroquinolones. Today, however, it counts as a last-resort drug in human medicine for the treatment of “sepsis and pneumonia caused by extensive drug-resistant Gram-negative bacteria” [51,74], making its regular usage in animals controversial.

EMA’s Category B (Restrict) of the “Categorisation of antibiotics […]” includes the third- and fourth-generation cephalosporins, quinolones, and polymyxins for veterinary use. Compared with the other two categorisations, macrolides are placed only in the third Category C (Caution). The reasons for this decision are discussed in the reflection paper [30,75]: the definition of substances assigned to Category C states that “there are in general alternatives in human medicine in the EU but there are few alternatives in veterinary medicine for certain indications”. These indications are, for example, the treatment of *Lawsonia intracellularis* and *Mycoplasma* spp. and the “treatment of respiratory tract infections caused by bacteria that are resistant to alternatives in Category D” [76]. Whereas the EMA acknowledges that the usage of macrolides in animals may lead to increasing resistance to macrolides in *Campylobacter*, *Salmonella*, and other pathogens in humans, it cites studies on risk assessment suggesting that the usage of macrolides in animals poses a lower public health risk [77,78] than AMU in humans [4,79]. Furthermore, campylobacteriosis in humans does not usually require treatment, as it is mostly self-limiting and severe courses of infection often associate with co-existing diseases or geriatric patients [80]. However, other reports have documented high resistance levels to tylosin in bacteria from pigs, including zoonotic pathogens in several European countries [81,82,83]. Given the threat of resistance and the importance of macrolides for the treatment of some indications in children, in whom the administration of fluoroquinolones is not possible, the EMA reclassified this substance class from the previous “no restrictions on use” (Category D) to “caution” (Category C). In addition to these risk factors, this decision also considered that, depending on the disease, alternatives are available for prevention, metaphylaxis, and treatment—such as pleuromutilins, tetracyclines, lincosamides, and penicillins [51]—but placing macrolides in Category B would severely limit the availability of alternatives for those diseases.

With the created list, the EMA considers both human and animal health, complying with a One Health approach to combat AMR. Different reflection papers describe thorough scientific evaluations on critical antimicrobials, especially polymyxins and macrolides [74,84]. In addition to the mentioned update of macrolides, polymyxins were upgraded from Category C to B, because risk factors for public health through resistance development increased or new data became available. According to the EMA, this list can assist in creating guidelines at the national level while considering regional requirements.

After applying the three different lists of antimicrobial categorisations to our data, with the goal of analysing the consequences for treatment guidelines for veterinarians, the one created by the EMA—as a One Health approach—seems to be most appropriate, but still needs to be adjusted for national use.

Macrolides, defined as second-line antimicrobials by EMA, are widely used in veterinary medicine and play a major role in the treatment of animals (as shown in the literature and our data alike). Placing this class into a more restricted category (as per the WHO and WOAH) would put its use in a bad light and might lead to the use of other critical antimicrobials or might endanger animal health if less-effective antimicrobials are used instead.

National adaptations of the EMA list, to establish treatment guidelines for Germany—supporting veterinarians in forming their treatment decision—should include a subdivision by animal species and, even further, by production type. They should also consider the differences in the nature and incidence of infectious diseases and, therefore, the differences in the needed substances [40,51,53,54,58], which are also reflected by the different usage patterns in our study. Thus, our findings could assist to define first-, second-, and third-line antimicrobials for such (production-type-based) treatment guidelines in Germany.

According to the new regulation (EU) 2019/6, which came into force starting 28 January 2022 [13], criteria have to be determined to identify antimicrobial substances reserved for human use only, considering scientific-based recommendations from the EMA and other European agencies [85]. These criteria were fixed by Delegated Regulation (EU) 2021/1760 [86]. As a next step, on 16 February 2022, the EMA published a list of antimicrobials recommended to be reserved for human use only [87] based on evaluations in accordance with the previously fixed groups of criteria: “importance for human health, resistance transmission risk from animals to humans and non-essential need in veterinary medicine”. This list includes antimicrobials such as glycopeptides and penems, but does not include any substances licensed for use in veterinary medicine in the European Union, meaning all currently available treatment options are recommended to remain available. On 19 July 2022, the European Commission implemented Regulation (EU) 2022/1255, designating antimicrobials in accordance with Regulation (EU) 2019/6 (from 9 February 2023 onwards), considering the EMA’s advice [88]. The list of designated restricted antimicrobials will be reviewed continually and adapted accordingly to the requirements. In addition, the German government decides on an amendment of the Veterinary Medicinal Products Act, including a higher impact on the total TF if third- and fourth-generation cephalosporins, fluoroquinolones, or colistin are administered [89]. The coming years will show how the European and national regulations will affect the treatment of sick animals and AMU in general, as well as of critical antimicrobials.

## 4. Materials and Methods

### 4.1. VetCAb Scientific Monitoring System

The VetCAb project started in 2007 as a feasibility study to determine if it would be possible to monitor AMU in German livestock husbandry at the species level [26]. In 2011, a cross-sectional study followed, for which data on AMU were collected throughout Germany [31]. In 2013, the project continued as a longitudinal approach. For this, data collection occurred semi-annually via veterinarians or farmers, using an open cohort with ongoing recruitment, to balance out possible withdrawals [73,90,91].

The provided data originated from application and delivery forms (ADFs), which have been mandatory in Germany since 1975. Due to regulations, the treating veterinarian has to document any antimicrobial prescription to livestock [24,92], including a variety of information such as identity, number, and type of animals treated; drug name and dosage; treatment days; indication; and application form. Additionally, all participants were requested to report the animal capacity of the individual farms. After the pseudonymisation of all data captured, the study team entered the information into a database exclusively designed for this project.

For this investigation, the following four production types as documented on the ADFs were analysed separately [31]: piglets up to four weeks of age (average weight 4 kg), weaners from four weeks of age up to 25–30 kg (average weight 15 kg), fattening pigs up to six months of age and 110–120 kg (average weight 50 kg), and sows (average weight 200 kg). These categories by weight resemble those defined in the ESVAC reports [14,15], but were rescaled to the German production requirements [93]. Checks for completeness and pharmacological plausibility were conducted, as previously described [90]. We excluded ADFs on local administered antimicrobials due to the inaccurate dosing and the low quantity of this pharmaceutical form (<2.5%). Thus, we retained and further analysed the ADFs on parenteral and oral usage only.

### 4.2. Lists on Antimicrobial Classification

To investigate AMU by antimicrobial class, we applied three different international classification systems:

1. WHO [28]

The WHO lists antimicrobials that are critically important to human health (last updated in 2018). All antimicrobials are divided into three categories: “critically important antimicrobials” (CIA, fulfilling both criteria), “highly important antimicrobials” (HIA, fulfilling either criterion), and “important antimicrobials” (IA, fulfilling neither criterion). Further separation prioritises substances that need risk management strategies most urgently. Hence, the CIA category is split into two groups divided by three factors: “highest priority critically important antimicrobials” (HPCIA, fulfilling all three factors) and “high priority critically important antimicrobials” (fulfilling zero–two factors).

2. WOAH [29]

The WOAH set up a list according to veterinarian opinion on the necessity of antimicrobials for veterinary use and their alternatives. Although the wording is similar to that of the WHO, the meaning behind the wording is different. The established categories are “veterinary critically important antimicrobial agents” (VCIA, fulfilling both criteria), “veterinary highly important antimicrobial agents” (VHIA, fulfilling either criterion), and “veterinary important antimicrobial agents” (VIA, fulfilling neither criterion).

3. EMA [30]

The current classification (last updated in 2019) consists of four categories (A–D) and includes only antimicrobial classes and substances authorised for use in humans and/or veterinary medicine in the EU. Category A (Avoid) includes antimicrobials not authorised for use in food-producing animals, whereas Category B (Restrict) includes the HPCIAs—except macrolides—and administration is limited to cases when no alternatives in a lower category are available to minimise the risk to (human) public health. Category C (Caution) mostly includes substances for which alternatives in human medicine exist but none or few are available for some indications in veterinary medicine. Category D (Prudence) encompasses antimicrobial substances considered to have a relatively low impact on human health—but which nevertheless have potential implications for the development of resistance, especially due to co-selection—and which still need to be administered only when necessary and always responsibly.

According to the ESVAC reports, the participating countries documented 104 substances in total [14], of which 69 were sold in Germany (BVL report, “Abgabenmengen-erfassung”) [44,94,95], whereas veterinarians participating in the VetCAb project used 36 substances for treating pigs. Table 5 contains a list of substances and classes used with pigs in the VetCAb study between 2013 and 2020 categorised by the WHO, WOAH, and EMA [28,29,30].

### 4.3. Statistical Evaluation

To quantify AMU, we calculated the treatment frequency (TF) as described by Merle et al. [26] using the number of used daily doses (UDDs):

(1) $$\mathrm{UDD}=\frac{\;\sum \mathrm{active}\;\mathrm{substance}\;(\mathrm{mg})}{\sum \mathrm{animals}\;\mathrm{treated}\;\times \;\sum \mathrm{treatment}\;\mathrm{days}\;\times \;\mathrm{animal}\;\mathrm{weight}\;\left(\mathrm{kg}\right)}$$

Formula (1) considers the amount of active substances used (in milligrams), the number of animals treated, the treatment days, and the current animal weight during therapy (in kilograms). The number of active substances can vary between the different VMPs used. If pharmaceuticals or treatments contain more than one different active compound, they contribute to the calculation with a value of two, or more. For one-shot and long-acting products, we used the number of effective days documented by the veterinarians as treatment days for the calculations.

In line with the calculation of the UDD, we calculated the total TF, which describes the average number of days that all animals within a stock are treated with any antimicrobial in a certain period [96,97,98]:

(2) $$\mathrm{TF}=\frac{\sum^{\;}\mathrm{active}\;\mathrm{substance}\;\mathrm{for}\;\mathrm{every}\;\mathrm{active}\;\mathrm{compound}\;\left(\mathrm{mg}\right)}{\mathrm{farm}\;\mathrm{size}\;\times \;\mathrm{animal}\;\mathrm{weight}\;\left(\mathrm{kg}\right)\;\times \;\mathrm{UDD}\left(\frac{\mathrm{mg}}{\mathrm{kg}}\right)}\;=\frac{\sum \mathrm{animals}\;\mathrm{treated}\;\times \;\sum \mathrm{treatment}\;\mathrm{days}\;\times \;\sum \mathrm{active}\;\mathrm{compounds}}{\mathrm{farm}\;\mathrm{size}\;}$$

We used Formula (2) for our calculations, in line with the well-known treatment incidence (TI) in other monitoring programs [32,33,34], but we used the UDD and the body weight under treatment instead of the defined daily dose (DDD) and a standardised body weight for the calculation of TI.

We used the number of livestock places documented to indicate farm size [20,90]. In case the number of livestock places for piglets was not provided, we calculated this value by multiplying the number of livestock places for sows by 10.25, which represents the average number of piglets per litter in Germany [99].

Furthermore, we calculated the TF for individual substances, substance classes, and combinations of these. To calculate an average value for a certain substance (j) as a percentage of the total TF and consider the effect of different farm sizes (N_i_ = number of animals kept on a certain farm (i)), we adjusted the equation as follows:

(3) $${\mathrm{weighted}\;\mathrm{TF}(\%)}_{\mathrm{substance}\;j}=\frac{\sum_{i}N_{i}{\;\times \;\mathrm{TF}}_{\mathrm{substance}\;j}}{\sum_{i}N_{i}{\;\times \;\mathrm{TF}}_{i}}\times \;100$$

Using the TF and the weighted TF (TF_w%_) in percent per half year, we separately calculated the usage of antimicrobial classes and single substances for every type of production, and we divided by classification of antimicrobial substances in accordance with the WHO, WOAH, and EMA lists.

Lastly, we calculated the weighted TF for the eight main indications defined in our study: central nervous system, intestinal disease, joint disease, mastitis, urogenital diseases, respiratory diseases, skin diseases, and “other” diseases. For this, we adjusted Formula (3) as follows:

(4) $${\mathrm{weighted}\;\mathrm{TF}(\%)}_{\mathrm{indication}}=\frac{\sum_{i}N_{i}{\;\times \;\mathrm{TF}}_{\mathrm{indication}}}{\sum_{i}N_{i}{\;\times \;\mathrm{TF}}_{i}}\times \;100$$

All statistical calculations were performed with SAS 9.4 TS level 1M5 (SAS Institute Inc., Cary, NC, USA).

## 5. Conclusions

After careful consideration of the existing lists and their main focuses—being mainly humans for the WHO, mainly animals for the WOAH, and the EMA trying to combine both—for now, we consider the EMA list to be the most suitable for application to create national treatment guidelines in veterinary medicine in Germany, based on its One Health approach and risk evaluation of macrolide usage. However, the use of macrolides remains a much-discussed topic and needs further research regarding antimicrobial resistance and alternative treatment options in animals. Consequently, antimicrobial stewardship and prudent use remains of utmost importance.

With regard to the goal of AMU reduction, we observed notable decreases in the total amount of antimicrobials, which was also reflected in reductions in the use of cephalosporins and fluoroquinolones. Encouragingly, the use of polymyxins (mainly colistin)—recently categorised as critical antimicrobials—decreased considerably. To continue this trend, the emphasis on the use of prevention strategies and alternative treatment methods must be increased, which requires a better understanding of when exactly and for what specific indications veterinarians administer those antimicrobials, necessitating additional studies. With this, guidelines can be created that are more precisely adjusted to the needs of individual species and even production types, possibly limiting the use of critical antimicrobials to specific indications, reducing AMU for those antimicrobials even further.

## Figures and Tables

**Table 1:** Weighted treatment frequency (%) by WHO, WOAH, and EMA classification for piglets in VetCAb study per half year from 2013-1 to 2020-2.

Class	13-1	13-2	14-1	14-2	15-1	15-2	16-1	16-2	17-1	17-2	18-1	18-2	19-1	19-2	20-1	20-2
WHO Classification																
HPCIA	28.3	30.3	36.7	37.1	45.9	36.2	21.7	27.2	28.1	32.6	35.3	37.5	42.8	44.8	22.4	49.8
CIA	33.6	37.6	42.1	43.2	40.5	51.0	58.8	57.8	52.5	50.7	40.9	44.4	45.2	38.4	53.1	46.5
HIA	37.4	31.9	21.1	19.5	13.4	12.4	18.9	14.7	19.1	16.1	23.4	17.5	11.4	16.4	24.0	3.4
IA	0.7	0.3	0.1	0.1	0.3	0.3	0.5	0.3	0.4	0.6	0.4	0.6	0.6	0.4	0.5	0.2
WOAH Classification																
VCIA	83.5	87.6	83.3	87.6	91.0	94.9	99.7	99.8	99.3	99.5	99.4	98.9	95.9	96.0	95.5	94.2
VHIA	16.5	12.4	16.7	12.4	9.0	5.1	0.3	0.2	0.7	0.5	0.6	1.1	4.1	4.0	4.5	5.8
EMA Classification																
B	21.1	20.1	24.0	19.4	20.5	13.7	12.8	14.5	13.8	15.9	9.5	5.5	7.4	10.4	11.6	15.0
C	21.0	22.1	24.1	25.3	35.2	33.3	26.4	25.7	33.6	32.1	37.9	45.2	45.5	50.3	32.7	37.3
D	57.9	57.8	51.8	55.3	44.3	53.1	60.8	59.8	52.5	52.0	52.7	49.2	47.1	39.3	55.7	47.6

WHO: World Health Organisation, HPCIA: highest priority critically important antimicrobials, CIA: critically important antimicrobials, HIA: highly important antimicrobials, IA: important antimicrobials; WOAH: World Organisation for Animal Health, VCIA: veterinary critically important antimicrobials, VHIA: veterinary highly important antimicrobials; EMA: European Medicine Agency, B: restrict, C: caution, D: prudence; 13-1 to 20-2: 2013-1 to 2020-2.

**Table 2:** Weighted treatment frequency (%) by WHO, WOAH, and EMA classification for sows in VetCAb study per half year from 2013-1 to 2020-2.

Class	13-1	13-2	14-1	14-2	15-1	15-2	16-1	16-2	17-1	17-2	18-1	18-2	19-1	19-2	20-1	20-2
WHO Classification																
HPCIA	44.1	12.9	19.5	23.1	19.8	21.3	15.6	15.3	10.2	10.6	10.2	7.8	17.3	9.9	8.1	8.7
CIA	12.0	18.2	26.3	26.6	20.5	22.7	32.7	20.9	15.5	18.8	22.2	24.3	31.8	53.9	50.7	59.2
HIA	42.5	67.3	52.7	49.0	57.7	54.5	47.4	61.7	72.9	69.6	66.9	66.2	50.1	35.1	40.6	31.7
IA	1.4	1.6	1.4	1.4	2.0	1.5	4.4	2.1	1.4	1.0	0.8	1.7	0.9	1.1	0.5	0.4
WOAH Classification																
VCIA	95.1	96.7	88.5	86.6	97.4	98.1	93.1	97.6	98.3	98.7	99.2	98.3	99.1	98.9	99.4	99.6
VHIA	4.9	3.3	11.5	13.4	2.6	1.9	6.9	2.4	1.7	1.3	0.8	1.7	0.9	1.1	0.6	0.4
EMA Classification																
B	12.6	10.0	17.6	17.7	12.0	14.6	13.8	12.8	7.6	8.4	7.4	2.7	9.8	4.7	3.0	4.0
C	34.1	9.3	3.7	9.0	14.1	9.8	9.3	7.2	5.5	4.2	4.6	8.7	11.8	8.5	8.5	6.6
D	53.2	80.7	78.6	73.3	73.9	75.6	76.8	80.0	86.9	87.4	87.9	88.6	78.4	86.7	88.5	89.4

WHO: World Health Organisation, HPCIA: highest priority critically important antimicrobials, CIA: critically important antimicrobials, HIA: highly important antimicrobials, IA: important antimicrobials; WOAH: World Organisation for Animal Health, VCIA: veterinary critically important antimicrobials, VHIA: veterinary highly important antimicrobials; EMA: European Medicine Agency, B: restrict, C: caution, d: prudence; 13-1 to 20-2: 2013-1 to 2020-2.

**Table 3:** Weighted treatment frequency (%) by WHO, WOAH, and EMA classification for weaners the VetCAb study per half year from 2013-1 to 2020-2.

Class	13-1	13-2	14-1	14-2	15-1	15-2	16-1	16-2	17-1	17-2	18-1	18-2	19-1	19-2	20-1	20-2
WHO Classification																
HPCIA	39.0	37.0	37.3	39.5	34.4	31.5	29.4	23.5	28.4	28.3	30.8	27.7	33.1	29.0	28.9	24.1
CIA	34.2	35.5	36.9	38.3	38.4	42.2	39.8	43.7	44.0	47.0	42.3	45.1	35.6	46.9	45.4	47.4
HIA	26.1	27.4	25.4	21.5	26.4	24.5	28.5	30.3	26.1	22.2	23.1	24.8	30.4	22.3	24.2	27.5
IA	0.7	0.1	0.4	0.7	0.9	1.8	2.2	2.5	1.5	2.5	3.8	2.3	0.9	1.9	1.6	1.0
WOAH Classification																
VCIA	65.7	63.9	65.4	63.0	76.6	74.1	78.9	78.1	72.9	72.0	71.9	76.3	71.7	74.2	74.5	79.8
VHIA	34.3	36.1	34.6	37.0	23.4	25.9	21.1	21.9	27.1	28.0	28.1	23.7	28.3	25.8	25.5	20.2
EMA Classification																
B	33.5	34.3	34.1	36.7	23.5	25.2	20.3	20.2	26.9	26.6	25.2	22.0	28.3	24.9	25.0	24.1
C	11.0	7.1	6.6	5.3	14.7	10.1	14.9	8.9	7.4	7.5	11.9	9.0	8.8	12.5	8.1	47.4
D	55.5	58.6	59.4	58.0	61.8	64.7	64.8	71.0	65.7	65.8	62.8	69.0	62.9	62.6	66.9	27.5

WHO: World Health Organisation, HPCIA: highest priority critically important antimicrobials, CIA: critically important antimicrobials, HIA: highly important antimicrobials, IA: important antimicrobials; WOAH: World Organisation for Animal Health, VCIA: veterinary critically important antimicrobials, VHIA: veterinary highly important antimicrobials; EMA: European Medicine Agency, B: restrict, C: caution, D: prudence; 13-1 to 20-2: 2013-1 to 2020-2.

**Table 4:** Weighted treatment frequency (%) by WHO, WOAH, and EMA classification for fattening pigs in VetCAb study per half year from 2013-1 to 2020-2.

Class	13-1	13-2	14-1	14-2	15-1	15-2	16-1	16-2	17-1	17-2	18-1	18-2	19-1	19-2	20-1	20-2
WHO Classification																
HPCIA	25.3	27.9	27.8	25.3	19.9	22.2	21.3	17.6	15.8	28.0	19.3	14.3	19.4	15.2	19.5	26.9
CIA	26.6	31.2	33.5	30.5	31.3	32.1	36.5	36.8	30.8	34.0	32.3	34.3	31.5	32.7	27.8	28.3
HIA	38.2	34.4	33.6	36.1	42.5	38.5	30.2	36.9	39.8	30.5	36.3	34.5	32.7	38.3	41.1	34.1
IA	9.9	6.5	5.1	8.1	6.3	7.1	12.0	8.7	13.6	7.5	12.0	16.9	16.4	13.7	11.6	10.7
WOAH Classification																
VCIA	80.3	77.4	81.1	78.7	88.1	86.7	80.2	87.4	82.4	83.3	83.0	75.9	76.8	81.3	85.6	86.1
VHIA	19.7	22.6	18.9	21.3	11.9	13.3	19.8	12.6	17.6	16.7	17.0	24.1	23.2	18.7	14.4	13.9
EMA Classification																
B	9.2	15.1	13.6	13.4	5.8	6.8	9.5	6.2	6.0	9.2	6.2	6.1	6.7	4.2	2.9	3.3
C	28.6	22.1	21.4	21.1	24.0	24.9	26.7	22.1	25.9	30.8	29.4	31.1	33.7	27.8	30.9	36.5
D	62.2	62.9	65.0	65.6	70.1	68.3	63.7	71.7	68.1	59.9	64.4	62.9	59.6	68.0	66.2	60.2

WHO: World Health Organisation, HPCIA: highest priority critically important antimicrobials, CIA: critically important antimicrobials, HIA: highly important antimicrobials, IA: important antimicrobials; WOAH: World Organisation for Animal Health, VCIA: veterinary critically important antimicrobials, VHIA: veterinary highly important antimicrobials; EMA: European Medicine Agency, B: restrict, C: caution, D: prudence; 13-1 to 20-2: 2013-1 to 2020-2.

**Table 5:** List of substances including substance class used with pigs in VetCAb study between 2013 and 2020, as categorised by WHO, WOAH, and EMA [29,30,31].

Substance Class	Substance	WHO	WOAH	EMA
Aminoglycosides	Apramycin	CIA	VCIA	C
	Dihydrostreptomycin	CIA	VCIA	C
	Gentamicin	CIA	VCIA	C
	Neomycin	CIA	VCIA	C
	Paromomycin	CIA	VCIA	C
	Spectinomycin	IA	VCIA	D
Amphenicoles	Florfenicol	HIA	VCIA	C
Cephalosporins	Cefquinome	HPCIA	VCIA	B
	Ceftiofur	HPCIA	VCIA	B
Fluoroquinolones	Danofloxacin	HPCIA	VCIA	B
	Enrofloxacin	HPCIA	VCIA	B
	Marbofloxacin	HPCIA	VCIA	B
Lincosamides	Lincomycin	HIA	VHIA	C
Macrolides	Erythromycin	HPCIA	VCIA	C
	Gamithromycin	HPCIA	VCIA	C
	Tildipirosin	HPCIA	VCIA	C
	Tilmicosin	HPCIA	VCIA	C
	Tulathromycin	HPCIA	VCIA	C
	Tylosin	HPCIA	VCIA	C
	Tylvalosin	HPCIA	VCIA	C
Penicillins	Amoxicillin	CIA	VCIA	D
	Ampicillin	CIA	VCIA	D
	Benzylpenicillin	HIA	VCIA	D
	Penethamate	HIA	VCIA	D
Pleuromutilins	Tiamulin	IA	VHIA	C
Polymyxins	Colistin	HPCIA	VHIA	B
Sulfonamides	Sulfadiazine	HIA	VCIA	D
	Sulfadimethoxine	HIA	VCIA	D
	Sulfadimidine	HIA	VCIA	D
	Sulfadoxine	HIA	VCIA	D
	Sulfamethoxazole	HIA	VCIA	D
Tetracyclines	Chlortetracycline	HIA	VCIA	D
	Doxycycline	HIA	VCIA	D
	Oxytetracycline	HIA	VCIA	D
	Tetracycline	HIA	VCIA	D
Trimethoprim	Trimethoprim	HIA	VCIA	D

WHO: World Health Organisation, WOAH: World Organisation for Animal Health, EMA: European Medicine Agency, HPCIA: highest priority critically important antimicrobials, CIA: critically important antimicrobials, HIA: highly important antimicrobials, IA: important antimicrobials, VCIA: veterinary critically important antimicrobials, VHIA: veterinary highly important antimicrobials, B: restrict, C: caution, D: prudence.

## Data Availability

The data were collected on an individual basis from farmers and veterinary practitioners. Each participant provided written consent with the understanding that data would not be transferred to any third party. Therefore, data transfer to interested persons is not allowed without an additional formal contract. Data are available to qualified researchers who sign a contract with the University of Veterinary Medicine Hannover. This contract will include guarantees of the obligation to maintain data confidentiality in accordance with the provisions of the European General Data Protection Regulation and its supporting documents in Germany. Currently, there is no data access committee or another body who could be contacted for the data. However, for this purpose, a committee will be founded. This future committee will consist of the authors, as well as members of the University of Veterinary Medicine Hannover and members of the funding institution (Federal Institute for Risk Assessment). Interested cooperative partners who are able to sign a contract as described above may contact Lothar Kreienbrock (lothar.kreienbrock@tiho-hannover.de), Department of Biometry, Epidemiology and Information Processing, University of Veterinary Medicine Hannover, Bünteweg 2, 30559 Hannover.

## Supplementary Materials

The following supporting information can be downloaded at: https://www.mdpi.com/article/10.3390/antibiotics11121833/s1, Table S1: Treatment frequency for pigs in VetCAb study per production type and half year from 2013-1 to 2020-2; Table S2: Weighted treatment frequency per indication in piglets (%) in VetCAb study per half year from 2013-1 to 2020-2; Table S3: Weighted treatment frequency per indication in sows (%) in VetCAb study per half year from 2013-1 to 2020-2; Table S4: Weighted treatment frequency per indication in weaners (%) in VetCAb study per half year from 2013-1 to 2020-2; Table S5: Weighted treatment frequency per indication in fattening pigs(%) in VetCAb study per half year from 2013-1 to 2020-2; Table S6: Weighted treatment frequency per active substance in piglets (%) in VetCAb study per half year from 2013-1 to 2020-2; Table S7: Weighted treatment frequency per active substance in sows (%) in VetCAb study per half year from 2013-1 to 2020-2; Table S8: Weighted treatment frequency per active substance in weaners (%) in VetCAb study per half year from 2013-1 to 2020-2; Table S9: Weighted treatment frequency per active substance in fattening pigs (%) in VetCAb study per half year from 2013-1 to 2020-2; Figure S1: Weighted treatment frequency (%) by WHO, WOAH, and EMA classification for piglets in VetCAb study per half year from 2013-1 to 2020-2; Figure S2: Weighted treatment frequency (%) by WHO, WOAH, and EMA classification for sows in VetCAb study per half year from 2013-1 to 2020-2; Figure S3: Weighted treatment frequency (%) by WHO, WOAH, and EMA classification for weaners in VetCAb study per half year from 2013-1 to 2020-2; Figure S4: Weighted treatment frequency (%) by WHO, WOAH, and EMA classification for Fattening Pigs in VetCAb study per half year from 2013-1 to 2020-2.

## Abbreviations

ADF	Application and Delivery Form
AMR	Antimicrobial Resistance
AMU	Antimicrobial Usage
BVL	Federal Office of Consumer Protection and Food Safety
CIA	Critically Important Antimicrobials
DART	German Antibiotic Resistance Strategy
DDD	Defined Daily Dose
DIMDI	German Institute for Medicinal Documentation and Information
EMA	European Medicine Agency
ESVAC	European Surveillance of Veterinary Antimicrobial Consumption
HIA	Highly Important Antimicrobials
HPCIA	Highest Priority Critically Important Antimicrobials
IA	Important Antimicrobials
PCU	Population Correction Unit
TI	Treatment Incidence
TF	Treatment Frequency
TF_w%_	Weighted TF in percent
UDD	Used Daily Dose
VetCAb	Veterinary Consumption of Antibiotics
VCIA	Veterinary Critically Important Antimicrobials
VHIA	Veterinary Highly Important Antimicrobials
VIA	Veterinary Important Antimicrobials
VMP	Veterinary Medicinal Product
WHO	World Health Organisation
WOAH/OIE	World Organisation for Animal Health
