# Supplementary material for: Use of Antimicrobials by Class in Pigs in Germany—A Longitudinal Description Considering Different International Categorisation Systems
Source: Antibiotics (Basel). 2022 Dec 16;11(12):1833. doi: 10.3390/antibiotics11121833 (PMC9774131; doi:10.3390/antibiotics11121833)
Supplement: Supplementary file 1 [file antibiotics-11-01833-s001.zip › antibiotics-2070430-supplementary.pdf]

Supplementary Materials

Table S1. Treatment frequency for pigs in VetCAb study per production type and half year from 2013-1 to 2020-2.

Production Type	Half-Year	Holdings	Treatment Frequency						Antimicrobial Usage yes		no	
			Min	QRange	Lower Quartile	Median	Upper Quartile	Max	N Holdings	%	N Holdings	%
Piglet	2013-1	145	-	8.5	1.4	4.0	9.9	62.6	133	91.7	12	8.3
	2013-2	139	-	5.1	0.6	2.7	5.6	67.7	117	84.2	22	15.8
	2014-1	173	-	4.8	0.5	2.1	5.3	97.5	144	83.2	29	16.8
	2014-2	194	-	4.2	0.3	1.8	4.5	48.6	154	79.4	40	20.6
	2015-1	250	-	6.9	0.3	2.4	7.2	170.7	195	78.0	55	22.0
	2015-2	248	-	5.7	0.2	1.8	5.8	137.4	192	77.4	56	22.6
	2016-1	147	-	11.2	0.2	2.1	11.4	251.0	115	78.2	32	21.8
	2016-2	150	-	9.9	-	1.7	9.9	234.6	111	74.0	39	26.0
	2017-1	169	-	10.8	0.1	2.3	10.9	190.2	131	77.5	38	22.5
	2017-2	170	-	10.3	0.1	1.9	10.3	174.4	128	75.3	42	24.7
	2018-1	177	-	11.3	-	2.6	11.3	289.6	130	73.4	47	26.6
	2018-2	178	-	10.6	-	2.3	10.6	367.8	117	65.7	61	34.3
	2019-1	178	-	11.0	-	2.5	11.0	355.7	122	68.5	56	31.5
	2019-2	179	-	7.8	-	1.0	7.8	297.6	116	64.8	63	35.2
	2020-1	177	-	4.5	-	0.9	4.5	193.5	111	62.7	66	37.3
	2020-2	177	-	4.4	-	0.8	4.4	126.9	107	60.5	70	39.5
Sow	2013-1	145	-	5.2	0.2	1.0	5.4	63.0	123	84.8	22	15.2
	2013-2	139	-	4.4	0.2	1.1	4.6	50.4	116	83.5	23	16.5
	2014-1	173	-	3.4	0.4	1.3	3.8	369.9	152	87.9	21	12.1
	2014-2	194	-	4.6	0.3	1.2	4.9	127.6	169	87.1	25	12.9
	2015-1	258	-	2.7	0.2	0.9	2.9	50.5	208	80.6	50	19.4
	2015-2	256	-	1.9	0.1	0.6	2.0	38.2	198	77.3	58	22.7
	2016-1	155	-	2.2	0.1	0.7	2.3	27.2	121	78.1	34	21.9
	2016-2	158	-	2.2	0.1	0.7	2.3	26.5	122	77.2	36	22.8
	2017-1	177	-	2.8	0.2	1.0	3.0	50.4	141	79.7	36	20.3
	2017-2	178	-	2.7	0.2	0.8	2.9	35.1	141	79.2	37	20.8
	2018-1	185	-	3.3	0.1	0.8	3.4	34.2	142	76.8	43	23.2
	2018-2	186	-	2.8	-	0.7	2.8	46.1	139	74.7	47	25.3
	2019-1	186	-	2.2	-	0.7	2.2	33.8	130	69.9	56	30.1
	2019-2	187	-	2.9	-	0.7	2.9	47.9	135	72.2	52	27.8
	2020-1	185	-	2.4	-	0.6	2.4	54.1	126	68.1	59	31.9
	2020-2	185	-	2.1	-	0.6	2.1	57.7	121	65.4	64	34.6
Weaner	2013-1	156	-	24.9	0.0	7.4	25.0	264.9	118	75.6	38	24.4
	2013-2	156	-	35.3	-	8.6	35.3	348.8	113	72.4	43	27.6
	2014-1	205	-	43.4	0.8	11.6	44.2	414.6	167	81.5	38	18.5
	2014-2	217	-	27.1	-	7.0	27.1	142.9	160	73.7	57	26.3
	2015-1	305	-	13.6	-	2.9	13.6	104.1	213	69.8	92	30.2
	2015-2	298	-	9.3	-	1.5	9.3	93.0	203	68.1	95	31.9
	2016-1	196	-	8.0	-	0.9	8.0	115.4	122	62.2	74	37.8
	2016-2	199	-	9.1	-	1.0	9.1	125.0	125	62.8	74	37.2
	2017-1	213	-	7.7	-	1.3	7.7	97.6	142	66.7	71	33.3
	2017-2	214	-	7.2	-	0.9	7.2	90.5	137	64.0	77	36.0
	2018-1	222	-	6.9	-	0.9	6.9	162.3	141	63.5	81	36.5

Production Type	Half-Year	Holdings	Treatment Frequency						Antimicrobial Usage yes		Antimicrobial Usage no	
			Min	QRange	Lower Quartile	Median	Upper Quartile	Max	N Holdings	%	N Holdings	%
	2018-2	223	-	6.3	-	0.7	6.3	121.1	134	60.1	89	39.9
	2019-1	223	-	6.5	-	0.7	6.5	55.0	132	59.2	91	40.8
	2019-2	223	-	5.0	-	0.3	5.0	42.3	131	58.7	92	41.3
	2020-1	221	-	4.7	-	0.3	4.7	71.6	131	59.3	90	40.7
	2020-2	221	-	5.7	-	0.3	5.7	99.8	128	57.9	93	42.1
Fattening Pig	2013-1	413	-	11.6	0.1	2.5	11.7	158.6	319	77.2	94	22.8
	2013-2	443	-	9.9	0.0	2.4	9.9	258.6	341	77.0	102	23.0
	2014-1	545	-	7.8	0.0	1.0	7.9	285.6	410	75.2	135	24.8
	2014-2	559	-	6.6	0.1	0.7	6.7	280.0	440	78.7	119	21.3
	2015-1	768	-	2.5	-	0.2	2.5	52.3	492	64.1	276	35.9
	2015-2	759	-	2.5	-	0.2	2.5	44.1	497	65.5	262	34.5
	2016-1	434	-	2.4	-	0.1	2.4	40.5	261	60.1	173	39.9
	2016-2	436	-	2.4	-	0.1	2.4	143.8	267	61.2	169	38.8
	2017-1	466	-	2.2	-	0.2	2.2	150.9	300	64.4	166	35.6
	2017-2	468	-	2.8	-	0.2	2.8	119.7	310	66.2	158	33.8
	2018-1	480	-	2.2	-	0.1	2.2	45.9	295	61.5	185	38.5
	2018-2	481	-	1.7	-	0.1	1.7	163.8	286	59.5	195	40.5
	2019-1	482	-	2.0	-	0.1	2.0	163.6	292	60.6	190	39.4
	2019-2	482	-	1.4	-	0.1	1.4	93.1	300	62.2	182	37.8
	2020-1	479	-	1.7	-	0.1	1.7	108.7	294	61.4	185	38.6
	2020-2	479	-	1.3	-	0.1	1.3	202.5	291	60.8	188	39.2

Min: minimum, QRange: range between minimum and maximum, Max: maximum, N Holdings: number of farms.

Table S2. Weighted treatment frequency per indication in piglets (%) in VetCAb study per half year from 2013-1 to 2020-2.

Indication	13-1	13-2	14-1	14-2	15-1	15-2	16-1	16-2	17-1	17-2	18-1	18-2	19-1	19-2	20-1	20-2
Central Nervous System	0.1	0.0	0.0	0.0	0.0	.	.	.	.	0.0	1.8	.	.	.	.	0.0
Intestinal Disease	25.3	25.4	29.7	27.7	39.0	25.3	17.5	14.0	9.3	12.7	7.6	5.4	5.1	10.2	13.4	14.7
Joint Disease	5.9	6.5	13.2	11.7	25.8	30.3	29.6	35.3	51.3	48.4	28.2	23.6	20.3	13.9	18.8	2.6
Other Diseases	17.3	23.3	18.9	8.9	15.7	13.2	11.2	16.8	9.7	5.4	6.5	17.3	17.6	9.2	8.7	16.8
Urogenital Diseases	.	.	.	.	0.0	0.1	.	.	.	.	.	0.2	0.0	0.1	0.2	0.1
Respiratory Disease	47.7	42.4	34.2	33.3	16.3	20.9	27.6	23.4	21.5	25.6	52.1	51.8	55.8	44.7	20.2	46.8
Skin Disease	3.8	2.4	4.0	18.4	3.2	10.3	14.2	10.5	8.3	7.8	3.7	1.8	1.2	21.8	38.7	19.0
All	100.0	100.0	100.0	100.0	100.0	100.0	100.0	100.0	100.0	100.0	100.0	100.0	100.0	100.0	100.0	100.0

Table S3. Weighted treatment frequency per indication in sows (%) in VetCAb study per half year from 2013-1 to 2020-2.

Indication	13-1	13-2	14-1	14-2	15-1	15-2	16-1	16-2	17-1	17-2	18-1	18-2	19-1	19-2	20-1	20-2
Central Nervous System	.	.	0.0	.	0.8	.	.	.	0.0	.	0.0	.	.	.	.	.
Intestinal Disease	36.1	19.3	12.8	9.5	1.6	3.5	13.6	2.6	0.7	1.0	2.5	0.9	6.3	1.2	4.5	0.4
Joint Disease	1.9	1.1	2.4	3.9	3.9	4.0	6.3	2.9	4.9	2.6	3.7	2.7	6.8	25.9	19.3	20.4
Mastitis	.	.	.	.	.	.	.	.	.	.	.	.	.	.	0.0	0.0
Other Diseases	19.1	11.2	9.5	6.5	48.7	40.5	30.3	48.5	33.8	9.0	19.2	34.5	17.7	18.0	24.8	19.7
Urogenital Diseases	15.7	17.2	26.1	20.9	9.0	17.0	11.7	13.1	24.3	9.8	20.9	15.6	14.5	15.3	10.7	11.3
Respiratory Disease	27.0	51.1	38.6	48.7	34.8	34.2	37.8	32.5	36.1	77.4	53.4	46.1	54.7	26.7	27.6	23.6
Skin Disease	0.3	0.2	10.6	10.5	1.2	1.0	0.3	0.3	0.2	0.1	0.4	0.2	0.0	12.9	13.1	24.6
All	100.0	100.0	100.0	100.0	100.0	100.0	100.0	100.0	100.0	100.0	100.0	100.0	100.0	100.0	100.0	100.0

Table S4. Weighted treatment frequency per indication in weaners (%) in VetCAb study per half year from 2013-1 to 2020-2.

**Indication**	**13-1**	**13-2**	**14-1**	**14-2**	**15-1**	**15-2**	**16-1**	**16-2**	**17-1**	**17-2**	**18-1**	**18-2**	**19-1**	**19-2**	**20-1**	**20-2**
Central Nervous System	0.0	.	0.0	0.0	.	0.0	.	.	.	.	0.1	0.1	.	4.6	3.6	5.1
Intestinal Disease	44.6	43.2	41.1	43.6	31.4	27.3	30.6	31.6	38.4	33.3	30.5	20.7	45.1	34.8	28.9	24.3
Joint Disease	1.3	1.7	0.9	0.9	0.5	1.5	2.0	2.3	1.4	1.6	1.4	2.5	2.6	1.5	0.8	0.8
Other Diseases	2.6	9.0	4.4	7.5	3.9	4.8	0.3	0.7	3.6	0.1	1.3	2.4	2.7	2.7	6.7	13.5
Urogenital Diseases	.	.	.	.	.	.	.	0.5	.	.	.	.	.	.	0.0	0.0
Respiratory Disease	50.6	45.4	52.7	29.1	63.0	62.4	64.9	64.8	55.4	65.1	66.5	74.3	49.6	55.0	58.8	55.0
Skin Disease	1.0	0.7	0.8	18.9	1.2	3.9	2.2	0.1	1.2	0.1	0.3	0.0	0.0	1.5	1.3	1.3
All	100.0	100.0	100.0	100.0	100.0	100.0	100.0	100.0	100.0	100.0	100.0	100.0	100.0	100.0	100.0	100.0

Table S5. Weighted treatment frequency per indication in fattening pigs(%) in VetCAb study per half year from 2013-1 to 2020-2.

**Indication**	**13-1**	**13-2**	**14-1**	**14-2**	**15-1**	**15-2**	**16-1**	**16-2**	**17-1**	**17-2**	**18-1**	**18-2**	**19-1**	**19-2**	**20-1**	**20-2**
Central Nervous System	0.5	0.1	0.6	0.0	0.0	.	.	0.1	.	0.0	.	.	1.3	.	0.1	0.0
Intestinal Disease	38.5	35.0	32.8	27.6	40.3	29.9	31.7	30.9	16.7	28.7	18.5	25.4	30.0	19.6	25.8	22.1
Joint Disease	0.9	1.0	0.6	1.0	2.4	1.6	2.2	2.2	2.5	3.4	2.7	2.6	2.3	1.7	1.7	1.4
Other Diseases	4.1	12.4	3.1	4.2	1.1	1.0	3.0	1.7	3.7	0.4	1.5	4.8	5.4	0.9	2.3	5.9
Urogenital Diseases	.	.	.	.	.	.	.	.	.	.	.	.	.	.	.	0.0
Respiratory Disease	55.8	51.4	62.2	43.3	55.7	65.8	63.0	65.0	77.0	67.5	77.2	67.1	60.2	75.6	68.7	68.5
Skin Disease	0.3	0.2	0.8	23.9	0.6	1.7	0.1	0.2	0.1	0.0	0.1	0.2	0.7	2.2	1.3	2.1
All	100.0	100.0	100.0	100.0	100.0	100.0	100.0	100.0	100.0	100.0	100.0	100.0	100.0	100.0	100.0	100.0

Table S6. Weighted treatment frequency per active substance in piglets (%) in VetCAb study per half year from 2013-1 to 2020-2.

**Drug class**<br>Active Substance	**13-1**	**13-2**	**14-1**	**14-2**	**15-1**	**15-2**	**16-1**	**16-2**	**17-1**	**17-2**	**18-1**	**18-2**	**19-1**	**19-2**	**20-1**	**20-2**
**∑ Amphenicoles**	**0.2**	**0.8**	**0.0**	**0.4**	**0.4**	**0.3**	**0.1**	**0.4**	**0.2**	**.**	**0.1**	**0.3**	**0.1**	**0.0**	**0.1**	**0.1**
Florfenicol	0.2	0.8	0.0	0.4	0.4	0.3	0.1	0.4	0.2	.	0.1	0.3	0.1	0.0	0.1	0.1
**∑ Aminoglycosides**	**11.8**	**10.5**	**11.1**	**7.1**	**9.7**	**10.8**	**18.0**	**12.9**	**19.6**	**16.0**	**12.2**	**13.6**	**10.7**	**16.2**	**22.3**	**2.7**
Apramycin	0.4	0.4	0.8	0.5	0.3	0.4	0.1	0.1	0.2	0.2	0.1	0.1	0.2	1.0	0.6	1.0
Dihydrostreptomycin	9.2	7.2	6.0	5.3	8.4	9.0	15.8	11.1	17.3	14.4	11.2	12.0	8.7	13.9	19.7	0.0
Gentamicin	0.7	1.2	0.7	0.5	0.7	0.8	1.5	1.2	1.6	0.7	0.6	0.1	0.0	0.0	0.1	0.1
Neomycin	1.3	1.6	3.5	0.8	.	0.3	0.0	0.1	.	0.0	.	.	.	.	.	.
Paromomycin	.	.	.	.	.	.	.	.	.	.	.	0.8	1.2	0.9	1.5	1.4
Spectinomycin	0.2	0.1	0.1	0.1	0.2	0.3	0.5	0.3	0.4	0.6	0.4	0.6	0.6	0.4	0.5	0.2
**∑ Cephalosporins***	**2.3**	**2.3**	**2.3**	**2.8**	**5.8**	**4.3**	**5.7**	**7.4**	**2.8**	**6.7**	**3.8**	**2.6**	**1.0**	**1.7**	**0.4**	**3.4**
Cefquinome	0.4	0.1	0.4	0.1	0.2	0.2	0.2	0.4	0.3	0.2	0.1	0.0	0.1	0.1	0.2	0.3
Ceftiofur	1.9	2.2	1.9	2.6	5.6	4.1	5.5	7.1	2.5	6.5	3.7	2.6	0.9	1.6	0.2	3.1
**∑ Fluoroquinolones**	**4.3**	**6.2**	**5.4**	**4.3**	**5.8**	**4.3**	**6.9**	**6.9**	**10.4**	**8.7**	**5.0**	**1.8**	**2.3**	**4.6**	**6.7**	**5.9**
Danofloxacin	0.1	0.1	0.1	0.2	0.4	0.3	0.4	0.3	0.6	0.8	0.6	0.2	0.5	0.8	0.5	0.3
Enrofloxacin	4.0	5.9	5.0	4.0	5.3	3.9	6.5	6.4	9.7	7.8	4.4	1.5	1.8	3.8	6.2	5.5
Marbofloxacin	0.2	0.1	0.3	0.1	0.1	0.1	.	0.2	0.1	0.0	0.0	.	0.0	0.0	0.0	0.0
**∑ Lincosamides**	**1.6**	**0.7**	**0.3**	**0.0**	**0.0**	**0.0**	**0.0**	**.**	**.**	**.**	**.**	**.**	**0.0**	**.**	**0.0**	**.**
Lincomycin	1.6	0.7	0.3	0.0	0.0	0.0	0.0	.	.	.	.	.	0.0	.	0.0	.
**∑ Macrolides**	**7.2**	**10.2**	**12.7**	**17.7**	**25.3**	**22.6**	**8.9**	**12.7**	**14.2**	**16.7**	**25.8**	**32.0**	**35.4**	**34.4**	**10.8**	**34.8**
Gamithromycin	.	.	.	.	.	.	.	0.1	0.2	.	.	.	.	.	.	.
Tildipirosin	0.0	0.0	0.1	0.1	0.5	1.1	0.8	1.0	0.9	1.5	0.5	1.1	0.6	0.2	0.9	0.3
Tilmicosin	.	0.2	.	.	.	.	.	.	.	.	.	.	.	.	.	.
Tulathromycin	4.8	7.5	9.7	17.6	24.8	21.5	8.0	11.6	13.1	15.0	25.2	30.8	34.7	34.1	9.9	34.4
Tylosin	2.3	2.3	2.9	0.1	0.0	0.0	0.1	0.0	0.0	0.2	0.1	0.1	0.1	0.1	0.0	0.0
**∑ Penicillins**	**34.2**	**41.6**	**42.3**	**42.7**	**41.1**	**50.6**	**59.0**	**58.9**	**51.0**	**50.5**	**40.6**	**43.8**	**44.5**	**36.9**	**53.7**	**45.3**
Amoxicillin	22.1	26.7	31.0	36.2	31.1	40.6	41.4	45.2	33.3	35.3	29.0	31.4	35.1	22.6	31.3	44.1
Ampicillin	.	0.5	.	.	.	.	.	.	.	.	.	.	.	.	.	.
Benzylpenicilin	12.1	14.5	11.3	6.6	10.1	10.0	17.6	13.7	17.7	15.1	11.6	12.4	9.3	14.3	22.4	1.2
**∑ Pleuromutilins**	**0.4**	**0.1**	**.**	**0.1**	**0.1**	**.**	**.**	**.**	**.**	**.**	**.**	**.**	**.**	**.**	**.**	**.**
Tiamulin	0.4	0.1	.	0.1	0.1	.	.	.	.	.	.	.	.	.	.	.
**∑ Polymyxins**	**14.5**	**11.6**	**16.4**	**12.3**	**8.9**	**5.1**	**0.3**	**0.2**	**0.7**	**0.5**	**0.6**	**1.1**	**4.1**	**4.0**	**4.5**	**5.8**
Colistin	5.6	6.0	14.5	11.0	8.5	5.1	0.3	0.2	0.7	0.5	0.6	1.1	4.1	4.0	4.5	5.8
With ZnO	8.9	5.6	1.9	1.3	0.4	.	.	.	.	.	.	.	.	.	.	.
**∑ Sulfonamides**	**5.9**	**4.1**	**1.4**	**2.1**	**0.6**	**0.7**	**0.2**	**0.1**	**0.5**	**.**	**1.6**	**0.1**	**0.0**	**0.0**	**0.1**	**0.2**
Sulfadiazine	5.8	4.1	1.2	1.4	.	0.4	.	.	0.5	.	.	.	.	.	.	.
Sulfadimethoxine	.	.	.	.	0.3	.	.	.	.	.	1.6	.	.	.	.	.
Sulfadimidine	0.1	0.0	0.0	0.0	0.1	0.2	0.2	0.1	0.0	.	0.0	0.1	0.0	0.0	0.1	0.1
Sulfadoxine	0.1	0.0	0.3	0.6	0.2	0.0	.	.	.	.	.	.	.	0.0	0.0	0.0
**∑ Tetracyclines**	**11.6**	**7.8**	**6.5**	**8.3**	**1.8**	**0.8**	**0.7**	**0.4**	**0.1**	**0.9**	**8.5**	**4.8**	**2.0**	**2.0**	**1.3**	**1.8**
Chlortetracycline	0.8	0.7	1.9	6.9	0.1	0.1	.	.	.	0.0	.	.	.	0.0	.	.
Doxycycline	6.4	5.7	3.4	1.4	1.1	0.2	0.1	0.3	.	.	2.5	0.6	0.3	0.6	0.3	0.4
Oxytetracycline	0.1	0.0	0.1	0.0	0.4	0.4	0.6	0.2	0.1	0.8	6.0	4.2	1.6	1.4	1.0	1.4
Tetracycline	4.4	1.4	1.1	0.0	0.1	0.0	.	0.0	0.0	0.1	.	.	.	.	.	.
**∑ Trimethoprim**	**5.9**	**4.1**	**1.4**	**2.1**	**0.6**	**0.7**	**0.2**	**0.1**	**0.5**	**.**	**1.6**	**0.1**	**0.0**	**0.0**	**0.1**	**0.2**
Trimethoprim	5.9	4.1	1.4	2.1	0.6	0.7	0.2	0.1	0.5	.	1.6	0.1	0.0	0.0	0.1	0.2
**∑ TFw%**	**100**	**100**	**100**	**100**	**100**	**100**	**100**	**100**	**100**	**100**	**100**	**100**	**100**	**100**	**100**	**100**

*Cephalosporins: Cephalosporins of the third and fourth generation, TFw%: weighted treatment frequency in percent, 13-1 to 20-2: 2013-1 to 2020-2.

Table S7. Weighted treatment frequency per active substance in sows (%) in VetCAb study per half year from 2013-1 to 2020-2.

**Drug class**<br>Active Substance	**13-1**	**13-2**	**14-1**	**14-2**	**15-1**	**15-2**	**16-1**	**16-2**	**17-1**	**17-2**	**18-1**	**18-2**	**19-1**	**19-2**	**20-1**	**20-2**
**∑ Amphenicoles**	**0.2**	**0.1**	**0.2**	**0.2**	**1.0**	**1.3**	**1.0**	**1.8**	**0.5**	**0.5**	**0.5**	**1.9**	**2.6**	**1.4**	**0.8**	**1.2**
Florfenicol	0.2	0.1	0.2	0.2	1.0	1.3	1.0	1.8	0.5	0.5	0.5	1.9	2.6	1.4	0.8	1.2
**∑ Aminoglycosides**	**1.0**	**5.5**	**0.9**	**2.4**	**4.1**	**1.1**	**2.7**	**1.3**	**1.3**	**0.5**	**0.9**	**0.4**	**1.2**	**1.0**	**2.3**	**0.4**
Apramycin	0.1	.	0.0	0.1	0.1	.	.	.	.	.	.	.	.	.	2.0	.
Dihydrostreptomycin	0.1	4.7	0.2	1.2	1.7	.	0.7	.	0.8	.	0.4	.	0.5	0.8	.	.
Gentamicin	0.0	0.0	0.1	0.6	1.1	0.2	0.7	.	0.0	.	0.2	.	.	0.0	.	0.3
Neomycin	0.0	0.0	0.0	.	.	.	0.8	0.8	0.2	0.2	.	.	0.4	.	.	.
Spectinomycin	0.8	0.8	0.6	0.6	1.2	0.9	0.4	0.5	0.2	0.3	0.3	0.4	0.3	0.1	0.3	0.2
**∑ Cephalosporins***	**2.1**	**1.9**	**1.9**	**1.8**	**4.3**	**3.0**	**1.9**	**2.3**	**1.6**	**1.6**	**1.3**	**0.7**	**1.2**	**2.7**	**0.7**	**2.6**
Cefquinome	1.8	1.6	1.7	1.4	2.6	2.4	1.7	2.2	1.6	1.5	1.0	0.5	1.2	0.7	0.7	0.7
Ceftiofur	0.3	0.3	0.2	0.4	1.7	0.6	0.2	0.1	0.0	0.1	0.3	0.2	.	1.9	.	1.9
**∑ Fluoroquinolones**	**7.9**	**6.4**	**5.6**	**4.1**	**7.5**	**11.4**	**9.3**	**10.2**	**5.7**	**6.8**	**6.1**	**2.0**	**8.6**	**2.0**	**2.2**	**1.5**
Danofloxacin	1.0	0.3	0.7	0.2	0.3	0.4	0.2	0.1	0.2	0.2	0.1	0.0	0.2	0.0	0.0	0.0
Enrofloxacin	6.0	5.3	3.6	2.7	4.2	7.9	6.9	6.8	4.0	4.6	4.3	1.1	1.2	1.2	1.4	0.7
Marbofloxacin	0.9	0.7	1.2	1.2	3.0	3.1	2.1	3.3	1.4	1.9	1.7	0.9	7.2	0.8	0.7	0.7
**∑ Lincosamides**	**1.6**	**0.8**	**0.6**	**0.9**	**1.6**	**1.1**	**0.3**	**0.5**	**0.2**	**0.5**	**0.3**	**0.4**	**0.3**	**0.2**	**0.3**	**0.2**
Lincomycin	1.6	0.8	0.6	0.9	1.6	1.1	0.3	0.5	0.2	0.5	0.3	0.4	0.3	0.2	0.3	0.2
**∑ Macrolides**	**31.5**	**2.9**	**1.9**	**5.3**	**7.7**	**6.7**	**1.8**	**2.5**	**2.6**	**2.2**	**2.7**	**5.1**	**7.5**	**5.2**	**5.1**	**4.7**
Erythromycin	0.0	.	.	.	0.0	.	.	.	.	.	.	.	.	.	.	.
Gamithromycin	.	.	.	.	.	.	.	.	.	.	.	.	.	.	0.0	.
Tildipirosin	0.0	0.0	0.0	.	1.1	.	0.3	0.3	.	0.1	0.1	0.1	.	0.0	0.0	0.0
Tilmicosin	.	.	.	.	.	.	.	.	.	.	.	.	.	.	0.6	.
Tulathromycin	0.7	0.3	0.8	3.7	5.4	5.3	1.2	1.9	2.0	1.3	2.3	3.9	7.0	4.8	3.6	4.5
Tylosin	30.8	2.6	1.0	1.6	1.2	1.4	0.2	0.3	0.6	0.8	0.4	1.1	0.5	0.4	0.9	0.2
**∑ Penicillins**	**13.9**	**20.2**	**28.8**	**27.7**	**21.2**	**23.9**	**31.7**	**21.0**	**16.0**	**19.5**	**23.1**	**26.1**	**32.6**	**54.5**	**49.6**	**60.5**
Amoxicillin	11.8	13.5	26.0	24.8	17.6	22.5	30.3	20.1	14.5	18.6	21.6	24.3	30.9	53.0	48.7	58.9
Benzylpenicilin	2.1	6.7	2.7	3.0	3.6	1.4	1.4	0.9	1.5	0.9	1.5	1.8	1.8	1.4	0.9	1.6
Penethamate	.	0.0	0.0	0.0	.	.	.	.	.	.	.	.	.	.	.	.
**∑ Pleuromutilins**	**0.6**	**0.8**	**0.7**	**0.7**	**0.8**	**0.6**	**4.0**	**1.7**	**1.1**	**0.7**	**0.5**	**1.3**	**0.6**	**1.0**	**0.2**	**0.2**
Tiamulin	0.6	0.8	0.7	0.7	0.8	0.6	4.0	1.7	1.1	0.7	0.5	1.3	0.6	1.0	0.2	0.2
**∑ Polymyxins**	**2.7**	**1.7**	**10.1**	**11.8**	**0.1**	**0.2**	**2.6**	**0.3**	**0.3**	**.**	**.**	**.**	**0.0**	**.**	**0.1**	**0.0**
Colistin	0.4	.	1.3	7.2	.	0.1	2.6	0.3	0.3	.	.	.	0.0	.	0.1	0.0
With ZnO	2.3	1.7	8.8	4.6	0.1	0.1	.	.	.	.	.	.	.	.	.	.
**∑ Sulfonamides**	**7.6**	**9.0**	**8.3**	**8.3**	**16.4**	**15.6**	**16.5**	**20.2**	**22.3**	**16.4**	**15.3**	**17.6**	**15.5**	**8.9**	**10.6**	**7.6**
Sulfadiazine	6.1	8.5	6.9	6.9	11.7	9.3	9.9	14.0	14.8	0.7	0.0	.	1.0	.	.	.
Sulfadimethoxine	.	0.0	0.2	.	2.5	3.6	3.6	3.2	4.5	12.6	10.8	11.7	8.4	4.4	7.4	4.5
Sulfadimidine	1.3	0.4	1.0	1.2	1.9	2.5	2.9	3.0	3.0	3.1	4.5	5.9	6.1	4.4	3.2	3.1
Sulfadoxine	0.1	0.1	0.2	0.2	0.3	0.2	0.1	0.0	0.0	0.0	0.1	0.1	0.0	.	.	.
**∑ Tetracyclines**	**23.4**	**41.7**	**32.6**	**28.4**	**18.7**	**19.5**	**11.7**	**18.1**	**26.1**	**34.7**	**33.9**	**26.9**	**14.4**	**14.4**	**17.5**	**13.6**
Chlortetracycline	1.3	7.4	12.2	2.4	2.8	1.3	1.2	1.9	3.1	4.3	3.2	2.9	3.4	1.6	0.6	0.5
Doxycycline	4.5	4.3	7.9	4.6	10.6	10.1	7.3	14.4	18.9	29.0	26.8	22.5	9.8	12.2	16.5	12.7
Oxytetracycline	0.4	0.2	0.1	0.1	0.4	0.5	2.1	1.8	0.7	0.7	3.7	1.5	1.1	0.6	0.4	0.4
Tetracycline	17.3	29.9	12.4	21.3	4.8	7.6	1.1	.	3.5	0.8	0.2	.	.	.	.	.
**∑ Trimethoprim**	**7.6**	**9.0**	**8.3**	**8.3**	**16.4**	**15.6**	**16.5**	**20.2**	**22.3**	**16.4**	**15.3**	**17.6**	**15.5**	**8.9**	**10.6**	**7.6**
Trimethoprim	7.6	9.0	8.3	8.3	16.4	15.6	16.5	20.2	22.3	16.4	15.3	17.6	15.5	8.9	10.6	7.6
**∑ TFw%**	**100**	**100**	**100**	**100**	**100**	**100**	**100**	**100**	**100**	**100**	**100**	**100**	**100**	**100**	**100**	**100**

*Cephalosporins: Cephalosporins of the third and fourth generation, TFw%: weighted treatment frequency in percent, 13-1 to 20-2: 2013-1 to 2020-2.

Table S8. Weighted treatment frequency per active substance in weaners (%) in VetCAb study per half year from 2013-1 to 2020-2.

**Drug class**<br>Active Substance	**13-1**	**13-2**	**14-1**	**14-2**	**15-1**	**15-2**	**16-1**	**16-2**	**17-1**	**17-2**	**18-1**	**18-2**	**19-1**	**19-2**	**20-1**	**20-2**
**∑ Amphenicoles**	**0.1**	**0.0**	**0.1**	**0.1**	**0.5**	**0.2**	**0.7**	**1.0**	**0.2**	**0.3**	**0.3**	**0.4**	**0.8**	**0.5**	**0.2**	**0.1**
Florfenicol	0.1	0.0	0.1	0.1	0.5	0.2	0.7	1.0	0.2	0.3	0.3	0.4	0.8	0.5	0.2	0.1
**∑ Aminoglycosides**	**4.0**	**2.1**	**2.3**	**2.0**	**2.9**	**1.8**	**2.8**	**2.9**	**4.5**	**3.1**	**2.3**	**0.5**	**2.3**	**6.1**	**2.5**	**1.1**
Apramycin	.	0.0	0.1	.	0.2	.	.	0.3	0.4	.	.	.	.	.	.	.
Dihydrostreptomycin	0.2	0.1	0.0	.	0.1	0.1	0.0	0.0	0.0	0.0	0.0	0.0	.	0.0	.	.
Gentamicin	0.0	0.0	0.0	0.0	0.0	0.0	0.0	0.0	0.0	.	0.0	.	.	.	.	.
Neomycin	3.8	2.0	1.9	1.5	2.1	1.6	2.7	1.4	3.8	3.1	2.2	0.5	2.2	6.0	2.4	1.0
Spectinomycin	0.0	0.0	0.3	0.5	0.4	0.0	0.0	1.1	0.4	0.0	0.0	0.0	0.1	0.1	0.1	0.1
**∑ Cephalosporins***	**0.2**	**0.2**	**0.2**	**0.2**	**0.2**	**0.2**	**0.4**	**0.1**	**0.3**	**0.2**	**0.1**	**0.0**	**0.2**	**0.2**	**0.2**	**0.3**
Cefquinome	0.1	0.1	0.1	0.2	0.2	0.2	0.2	0.1	0.2	0.2	0.1	0.0	0.1	0.2	0.2	0.2
Ceftiofur	0.1	0.1	0.0	0.1	0.1	0.1	0.3	0.0	0.0	0.0	.	0.0	0.1	.	0.0	0.1
**∑ Fluoroquinolones**	**0.3**	**0.3**	**0.5**	**0.3**	**0.7**	**0.8**	**1.0**	**0.9**	**1.0**	**0.9**	**0.8**	**0.6**	**0.7**	**0.8**	**0.9**	**0.7**
Danofloxacin	0.0	0.0	0.0	0.0	0.0	0.1	0.1	0.0	0.1	0.1	0.1	0.0	0.1	0.1	0.0	0.1
Enrofloxacin	0.3	0.2	0.4	0.2	0.5	0.6	0.9	0.9	0.9	0.8	0.7	0.4	0.5	0.6	0.8	0.6
Marbofloxacin	0.0	0.0	0.0	0.0	0.2	0.0	.	.	0.0	0.0	0.1	0.2	0.1	0.1	0.1	0.0
**∑ Lincosamides**	**0.7**	**2.2**	**1.1**	**0.6**	**0.4**	**0.0**	**0.0**	**1.3**	**0.4**	**0.0**	**0.0**	**0.0**	**0.1**	**0.1**	**0.1**	**0.2**
Lincomycin	0.7	2.2	1.1	0.6	0.4	0.0	0.0	1.3	0.4	0.0	0.0	0.0	0.1	0.1	0.1	0.2
**∑ Macrolides**	**5.5**	**2.7**	**3.3**	**2.8**	**10.9**	**6.4**	**9.1**	**3.3**	**1.5**	**1.7**	**5.6**	**5.8**	**4.8**	**4.0**	**3.8**	**3.9**
Erythromycin	.	.	0.0	0.0	.	.	.	.	.	.	.	.	.	.	.	.
Tildipirosin	0.0	0.0	0.0	0.0	0.2	0.1	0.7	0.2	0.1	0.3	0.3	0.4	0.4	0.0	0.0	0.1
Tilmicosin	0.6	.	0.3	0.4	1.4	1.1	0.2	0.1	0.1	0.4	0.4	0.0	0.1	0.0	0.2	0.5
Tulathromycin	0.2	0.2	0.5	0.9	3.5	2.4	0.1	0.7	0.5	0.5	4.0	4.5	2.1	0.2	0.2	0.6
Tylosin	4.7	2.6	2.5	1.5	5.7	2.8	8.1	2.4	0.9	0.4	0.9	0.8	2.3	3.8	3.3	2.8
**∑ Penicillins**	**30.5**	**33.6**	**35.1**	**36.8**	**36.1**	**40.7**	**37.2**	**42.1**	**40.1**	**44.2**	**40.3**	**44.6**	**33.4**	**40.9**	**43.1**	**46.5**
Amoxicillin	30.3	33.4	34.9	36.7	35.9	40.5	37.1	41.9	39.8	43.9	40.1	44.6	33.4	40.8	43.0	46.4
Benzylpenicillin	0.2	0.2	0.2	0.1	0.2	0.2	0.1	0.2	0.3	0.3	0.2	0.0	0.0	0.1	0.1	0.1
**∑ Pleuromutilins**	**0.7**	**0.1**	**0.1**	**0.2**	**0.5**	**1.8**	**2.2**	**1.4**	**1.1**	**2.5**	**3.7**	**2.3**	**0.8**	**1.8**	**1.4**	**0.9**
Tiamulin	0.7	0.1	0.1	0.2	0.5	1.8	2.2	1.4	1.1	2.5	3.7	2.3	0.8	1.8	1.4	0.9
**∑ Polymyxins**	**32.9**	**33.9**	**33.4**	**36.1**	**22.6**	**24.1**	**18.9**	**19.1**	**25.6**	**25.5**	**24.3**	**21.3**	**27.4**	**23.9**	**23.9**	**19.2**
Colistin	14.7	16.3	16.8	25.5	16.7	21.1	13.8	18.9	25.3	24.8	24.2	21.3	27.4	18.8	20.2	13.3
Colistin with ZnO	18.2	17.6	16.6	10.6	5.9	3.0	5.1	0.2	0.3	0.7	0.1	.	.	5.1	3.7	5.9
**∑ Sulfonamides**	**1.9**	**1.4**	**2.0**	**1.4**	**3.9**	**1.4**	**3.2**	**2.3**	**1.5**	**0.8**	**0.7**	**0.4**	**1.2**	**0.5**	**3.3**	**3.4**
Sulfadiazine	1.8	1.4	2.0	1.4	3.5	1.4	3.2	2.3	1.4	0.2	0.5	0.1	0.3	.	2.3	3.2
Sulfadimethoxine	.	.	0.0	.	0.4	.	.	.	0.1	0.6	0.1	0.2	0.9	0.4	0.9	0.2
Sulfadimidine	0.0	0.0	0.0	0.0	0.0	0.0	0.0	0.0	0.0	0.0	.	0.0	0.0	0.1	0.1	0.0
Sulfadoxine	.	0.0	0.0	.	.	.	.	.	.	.	0.0	.	.	.	.	.
Sulfamethoxazole	0.1	.	.	.	.	.	.	.	.	.	.	.	.	.	.	.
**∑ Tetracyclines**	**21.2**	**22.1**	**20.0**	**17.8**	**17.5**	**21.2**	**21.3**	**23.3**	**22.2**	**20.0**	**21.2**	**23.6**	**27.1**	**20.6**	**17.2**	**20.4**
Chlortetracycline	2.0	1.6	1.7	2.0	4.1	1.6	.	0.5	3.4	2.4	2.4	1.8	2.9	0.6	1.5	1.5
Doxycycline	14.5	13.9	13.5	14.2	10.3	16.9	20.5	22.3	18.5	17.0	17.9	20.9	23.6	19.5	15.5	18.7
Oxytetracycline	0.1	0.0	0.0	0.0	0.2	0.2	0.6	0.5	0.3	0.3	0.7	0.9	0.6	0.5	0.2	0.2
Tetracycline	4.6	6.6	4.7	1.7	2.9	2.4	0.2	0.0	.	0.4	0.2	.	.	.	.	.
**∑ Trimethoprim**	**1.9**	**1.4**	**2.0**	**1.4**	**3.9**	**1.4**	**3.2**	**2.3**	**1.5**	**0.8**	**0.7**	**0.4**	**1.2**	**0.5**	**3.2**	**3.4**
Trimethoprim	1.9	1.4	2.0	1.4	3.9	1.4	3.2	2.3	1.5	0.8	0.7	0.4	1.2	0.5	3.2	3.4
**∑ TFw%**	**100**	**100**	**100**	**100**	**100**	**100**	**100**	**100**	**100**	**100**	**100**	**100**	**100**	**100**	**100**	**100**

*Cephalosporins: Cephalosporins of the third and fourth generation, TFw%: weighted treatment frequency in percent, 13-1 to 20-2: 2013-1 to 2020-2.

Table S9. Weighted treatment frequency per active substance in fattening pigs (%) in VetCAb study per half year from 2013-1 to 2020-2.

**Drug class**<br>Active Substance	**13-1**	**13-2**	**14-1**	**14-2**	**15-1**	**15-2**	**16-1**	**16-2**	**17-1**	**17-2**	**18-1**	**18-2**	**19-1**	**19-2**	**20-1**	**20-2**
**∑ Amphenicoles**	**0.2**	**0.1**	**0.1**	**0.1**	**1.1**	**0.8**	**0.8**	**0.9**	**0.8**	**0.7**	**1.0**	**1.8**	**1.6**	**1.1**	**1.1**	**1.4**
Florfenicol	0.2	0.1	0.1	0.1	1.1	0.8	0.8	0.9	0.8	0.7	1.0	1.8	1.6	1.1	1.1	1.4
**∑ Aminoglycosides**	**4.7**	**1.6**	**1.7**	**1.2**	**2.0**	**1.6**	**2.2**	**1.8**	**1.3**	**1.9**	**2.6**	**2.6**	**2.2**	**1.9**	**1.7**	**2.9**
Apramycin	0.1	.	.	.	.	.	.	.	.	.	0.0	.	.	.	.	.
Dihydrostreptomycin	0.1	0.0	0.0	0.1	.	.	.	.	.	.	.	.	.	0.0	.	.
Gentamicin	.	0.0	0.0	.	0.0	0.0	.	.	.	.	.	.	.	0.0	.	.
Neomycin	0.6	0.6	0.7	0.1	0.7	0.3	1.9	0.3	0.8	1.2	1.8	1.4	1.2	0.4	0.3	.
Spectinomycin	3.9	1.0	1.0	1.1	1.3	1.3	0.3	1.5	0.5	0.7	0.7	1.2	1.1	1.5	1.4	2.9
**∑ Cephalosporins***	**0.1**	**0.1**	**0.2**	**0.1**	**0.3**	**0.2**	**0.1**	**0.1**	**0.1**	**0.1**	**0.1**	**0.0**	**0.0**	**0.0**	**0.0**	**0.0**
Cefquinome	0.1	0.1	0.2	0.1	0.2	0.1	0.0	0.0	0.1	0.0	0.1	0.0	0.0	0.0	0.0	0.0
Ceftiofur	0.0	0.0	0.0	0.0	0.1	0.0	0.1	0.0	0.1	0.1	0.0	.	.	.	.	.
**∑ Fluoroquinolones**	**0.9**	**0.8**	**0.9**	**0.8**	**1.8**	**1.7**	**1.8**	**3.1**	**2.8**	**2.6**	**2.5**	**1.6**	**1.7**	**0.8**	**1.4**	**0.9**
Danofloxacin	0.3	0.4	0.3	0.3	0.5	0.4	0.6	0.8	1.1	1.2	0.5	0.4	0.7	0.5	0.8	0.4
Enrofloxacin	0.6	0.4	0.5	0.4	0.9	0.9	1.1	1.1	1.2	1.1	1.5	0.3	0.3	0.2	0.4	0.3
Marbofloxacin	0.0	0.1	0.1	0.1	0.4	0.5	0.1	1.2	0.6	0.3	0.6	0.9	0.7	0.2	0.2	0.2
**∑ Lincosamides**	**5.6**	**2.9**	**2.3**	**1.9**	**3.2**	**2.5**	**0.6**	**2.3**	**1.5**	**3.5**	**2.2**	**3.9**	**2.9**	**3.1**	**2.7**	**3.6**
Lincomycin	5.6	2.9	2.3	1.9	3.2	2.5	0.6	2.3	1.5	3.5	2.2	3.9	2.9	3.1	2.7	3.6
**∑ Macrolides**	**16.1**	**12.8**	**14.2**	**11.9**	**14.1**	**15.4**	**11.8**	**11.4**	**9.8**	**18.8**	**13.1**	**8.2**	**12.7**	**11.0**	**16.6**	**23.6**
Erythromycin	.	0.0	0.0	0.0	.	0.0	.	.	.	.	.	.	.	.	.	.
Tildipirosin	0.0	0.0	0.0	0.0	0.8	0.3	0.8	0.6	0.3	0.3	0.5	0.7	0.7	0.1	0.1	0.2
Tilmicosin	.	.	.	.	0.2	0.3	.	.	.	.	.	.	.	.	.	.
Tulathromycin	0.2	0.1	0.2	0.1	0.2	0.2	0.0	0.0	0.1	0.0	0.3	0.2	0.7	0.2	0.0	0.2
Tylosin	15.9	12.7	14.0	11.8	13.0	14.6	10.9	10.8	9.4	18.4	12.3	7.3	11.4	10.7	16.5	23.2
Tylvalosin	.	0.0	.	.	.	.	.	.	.	.	.	.	.	.	.	.
**∑ Penicillins**	**26.2**	**30.9**	**33.2**	**30.9**	**31.2**	**32.7**	**35.0**	**37.1**	**30.7**	**33.5**	**31.2**	**33.3**	**30.6**	**32.7**	**28.0**	**29.1**
Amoxicillin	25.8	30.5	32.6	30.4	30.6	31.8	34.6	36.5	30.1	32.8	30.5	32.9	30.3	32.3	27.5	28.3
Ampicillin	.	0.1	0.1	.	.	.	.	.	.	.	.	.	.	.	.	.
Benzylpenicilin	0.4	0.3	0.4	0.5	0.7	0.8	0.4	0.6	0.6	0.7	0.6	0.4	0.3	0.4	0.6	0.8
**∑ Pleuromutilins**	**5.9**	**5.6**	**4.1**	**7.0**	**4.9**	**5.8**	**11.7**	**7.3**	**13.0**	**6.7**	**11.2**	**15.8**	**15.3**	**12.2**	**10.2**	**7.9**
Tiamulin	5.9	5.6	4.1	7.0	4.9	5.8	11.7	7.3	13.0	6.7	11.2	15.8	15.4	12.2	10.2	7.9
**∑ Polymyxins**	**8.2**	**14.1**	**12.5**	**12.4**	**3.8**	**4.9**	**7.6**	**3.0**	**3.0**	**6.5**	**3.6**	**4.5**	**5.0**	**3.4**	**1.5**	**2.4**
Colistin	8.2	14.1	12.4	12.3	3.8	4.9	7.6	2.9	2.6	5.9	3.6	4.4	4.7	3.4	1.5	2.4
Colistin with ZnO	.	.	0.1	0.1	.	0.0	.	0.1	0.4	0.6	.	0.1	0.3	.	.	.
**∑ Sulfonamides**	**3.6**	**2.3**	**2.3**	**2.4**	**1.8**	**0.9**	**0.5**	**0.9**	**0.2**	**0.5**	**.**	**0.1**	**0.0**	**0.1**	**0.1**	**0.1**
Sulfadiazine	3.5	2.2	2.2	2.3	1.8	0.8	0.5	.	0.2	0.3	.	0.1	0.0	.	0.1	.
Sulfadimethoxine	0.0	0.0	.	.	.	.	.	0.9	.	0.1	.	.	.	0.1	.	.
Sulfadimidine	0.0	0.0	0.0	0.0	0.0	0.0	0.0	0.0	0.0	0.0	.	0.0	0.0	.	0.0	0.0
Sulfadoxine	0.0	0.0	0.0	0.0	0.0	0.1	.	.	0.0	0.0	.	.	.	0.0	0.0	0.1
**∑ Tetracyclines**	**24.9**	**26.5**	**26.3**	**28.8**	**33.9**	**32.6**	**27.4**	**31.3**	**36.4**	**24.7**	**32.5**	**28.2**	**27.9**	**33.5**	**36.4**	**28.1**
Chlortetracycline	8.1	9.0	10.0	5.8	5.1	3.1	0.1	.	0.1	0.2	0.3	0.8	0.1	.	0.1	2.3
Doxycycline	13.1	9.5	9.9	13.0	25.4	27.0	26.5	31.3	36.1	24.3	31.4	26.9	26.7	32.4	35.4	25.2
Oxytetracycline	0.1	0.1	0.1	0.0	0.2	0.2	0.2	0.0	0.1	0.1	0.3	0.5	1.1	1.1	0.9	0.7
Tetracycline	3.6	7.8	6.2	10.0	3.3	2.4	0.5	.	0.0	0.1	0.5	.	.	.	.	0.0
**∑ Trimethoprim**	**3.6**	**2.3**	**2.3**	**2.4**	**1.8**	**0.9**	**0.5**	**0.9**	**0.2**	**0.5**	**.**	**0.1**	**0.0**	**0.1**	**0.1**	**0.1**
Trimethoprim	3.6	2.3	2.3	2.4	1.8	0.9	0.5	0.9	0.2	0.5	.	0.1	0.0	0.1	0.1	0.1
**∑ TFw%**	**100**	**100**	**100**	**100**	**100**	**100**	**100**	**100**	**100**	**100**	**100**	**100**	**100**	**100**	**100**	**100**

*Cephalosporins: Cephalosporins of the third and fourth generation, TFw%: weighted treatment frequency in percent, 13-1 to 20-2: 2013-1 to 2020-2.

Figure S1. Weighted treatment frequency (%) by WHO, WOAH, and EMA classification for piglets in VetCAb study per half year from 2013-1 to 2020-2.

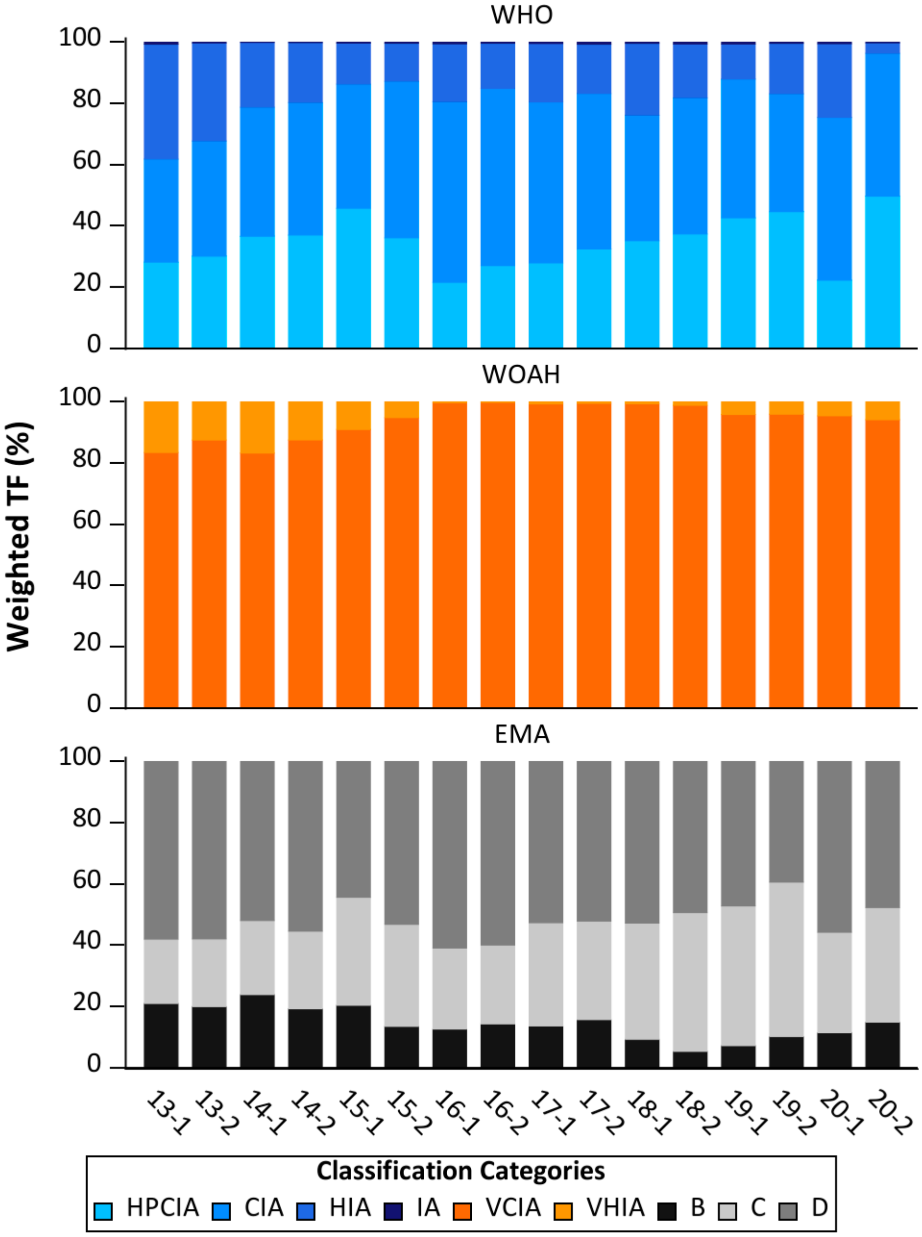

WHO: World Health Organisation, HPCIA: highest priority critically important antimicrobials, CIA: critically important antimicrobials, HIA: highly important antimicrobials, IA: important antimicrobials; WOAH: World Organisation for Animal Health, VCIA: veterinary critically important antimicrobials, VHIA: veterinary highly important antimicrobials; EMA: European Medicine Agency, B: restrict, C: caution, D: prudence; 13-1 to 20-2: 2013-1 to 2020-2.

Figure S2. Weighted treatment frequency (%) by WHO, WOAH, and EMA classification for sows in VetCAb study per half year from 2013-1 to 2020-2.

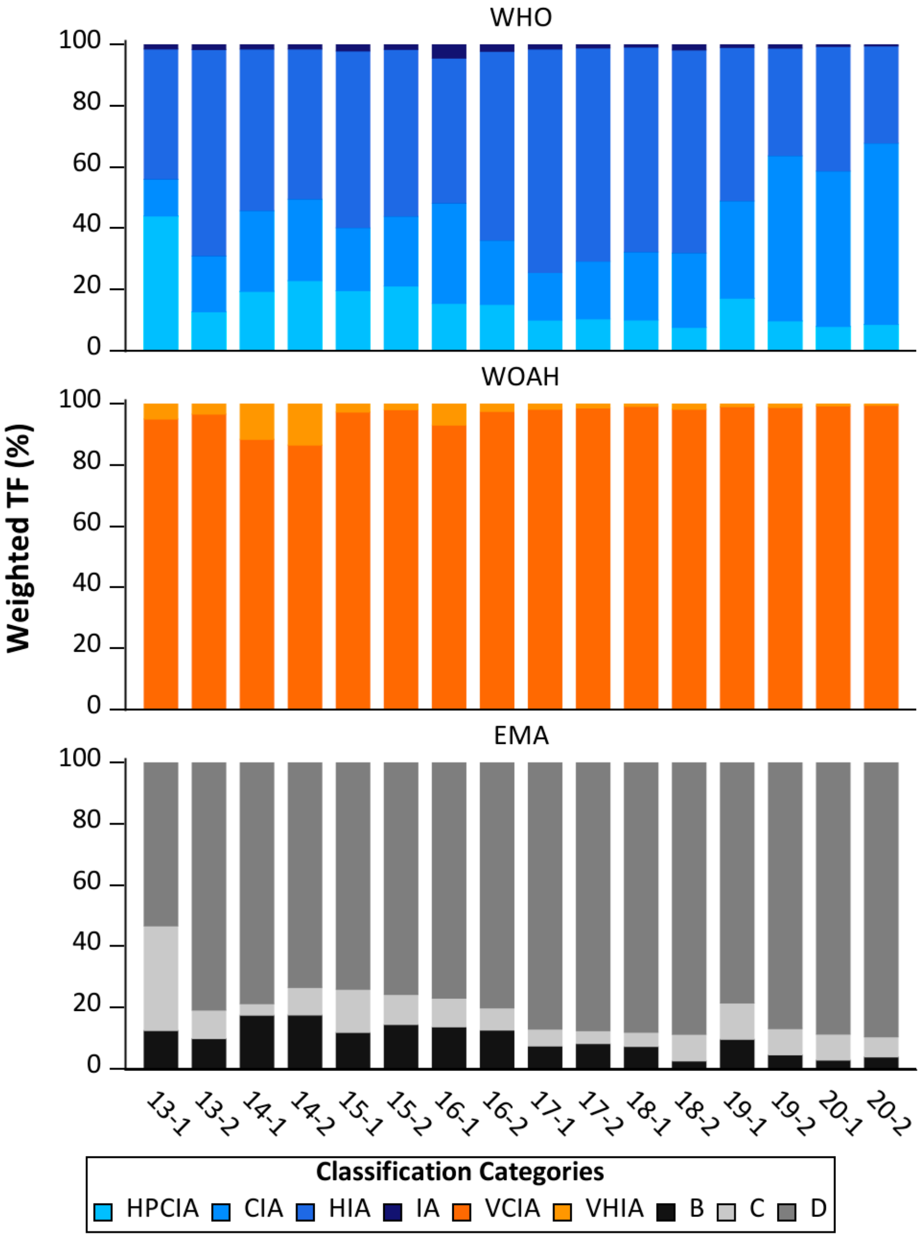

WHO: World Health Organisation, HPCIA: highest priority critically important antimicrobials, CIA: critically important antimicrobials, HIA: highly important antimicrobials, IA: important antimicrobials; WOAH: World Organisation for Animal Health, VCIA: veterinary critically important antimicrobials, VHIA: veterinary highly important antimicrobials; EMA: European Medicine Agency, B: restrict, C: caution, D: prudence; 13-1 to 20-2: 2013-1 to 2020-2.

Figure S3. Weighted treatment frequency (%) by WHO, WOAH, and EMA classification for weaners in VetCAb study per half year from 2013-1 to 2020-2.

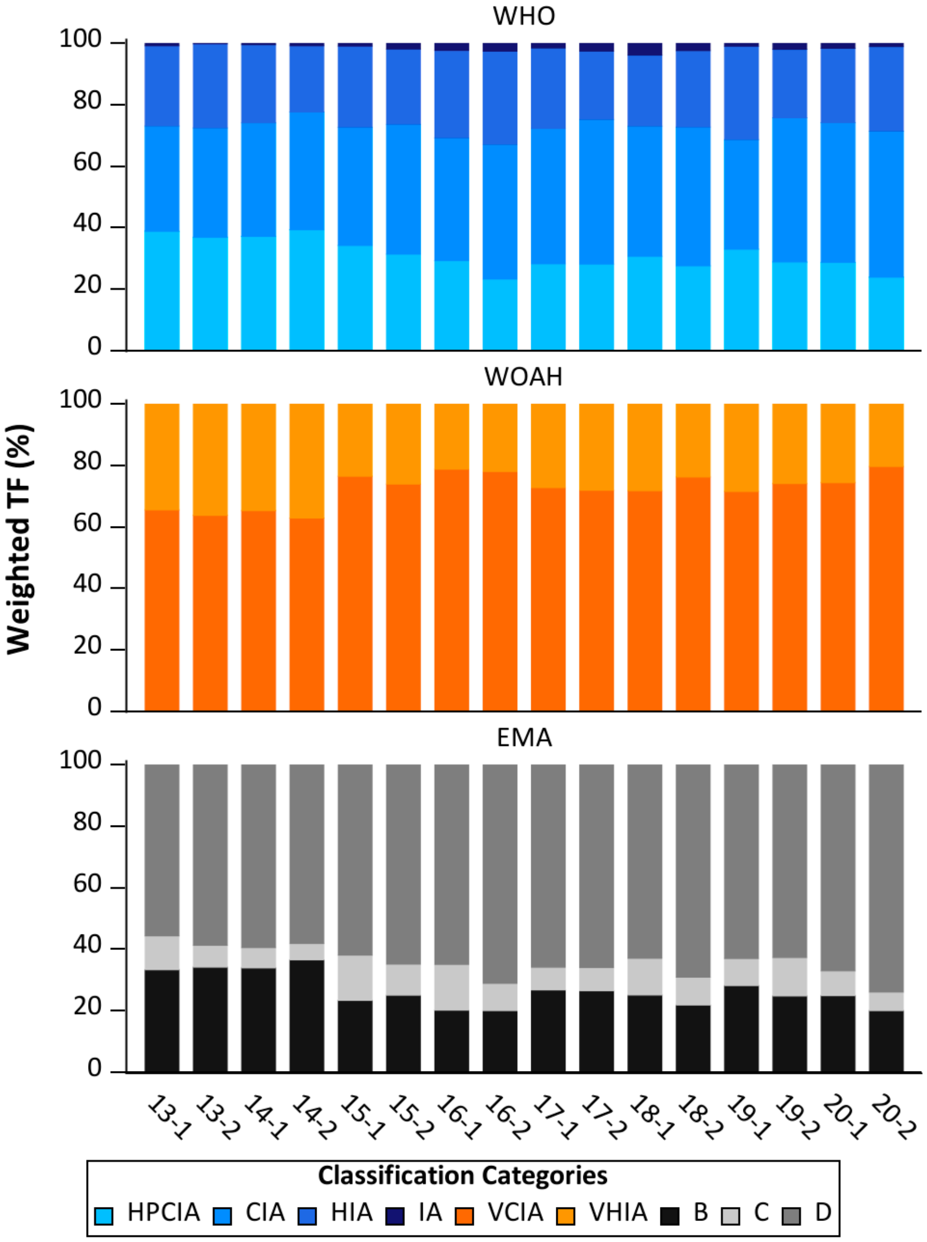

WHO: World Health Organisation, HPCIA: highest priority critically important antimicrobials, CIA: critically important antimicrobials, HIA: highly important antimicrobials, IA: important antimicrobials; WOAH: World Organisation for Animal Health, VCIA: veterinary critically important antimicrobials, VHIA: veterinary highly important antimicrobials; EMA: European Medicine Agency, B: restrict, C: caution, D: prudence; 13-1 to 20-2: 2013-1 to 2020-2.

Figure S4. Weighted treatment frequency (%) by WHO, WOAH, and EMA classification for Fattening Pigs in VetCAb study per half year from 2013-1 to 2020-2.

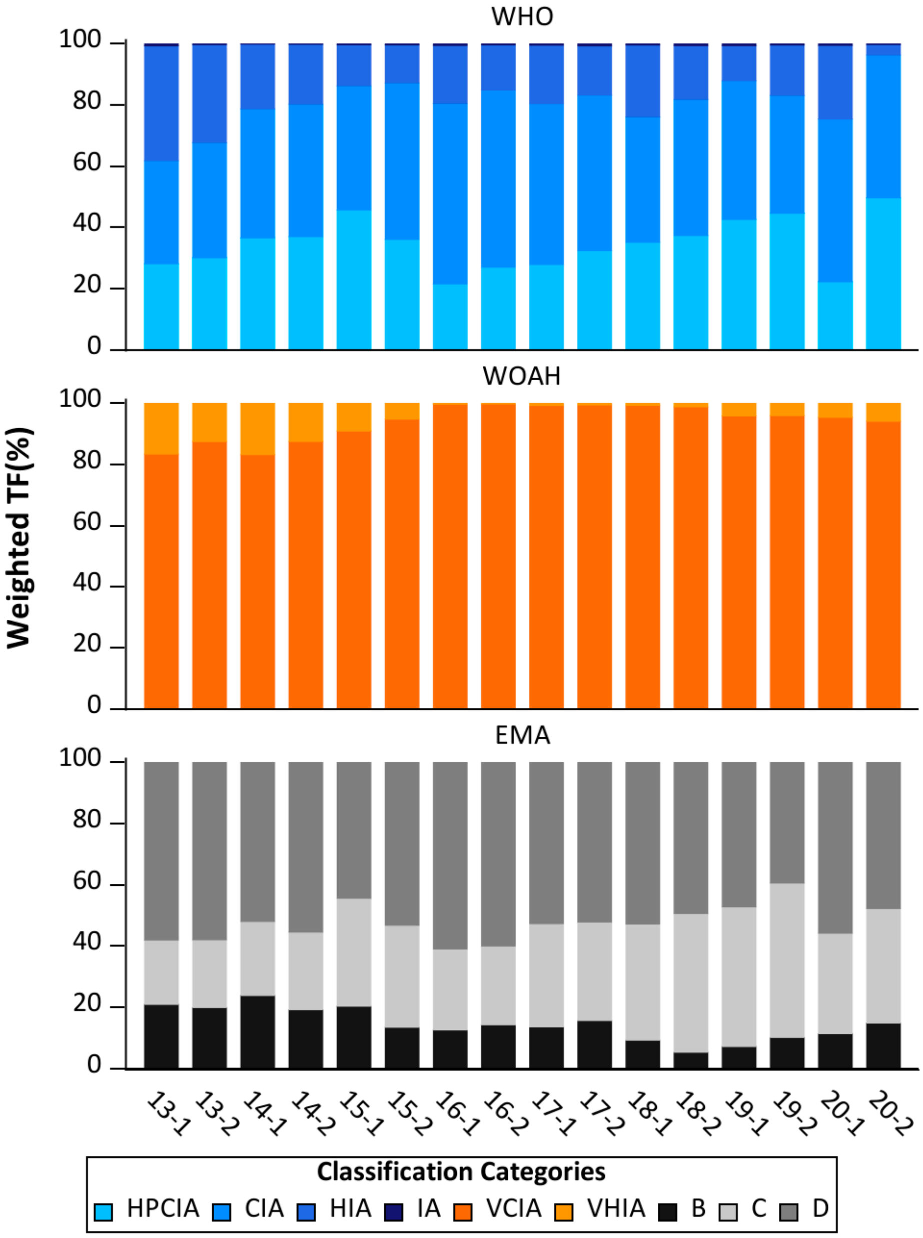

WHO: World Health Organisation, HPCIA: highest priority critically important antimicrobials, CIA: critically important antimicrobials, HIA: highly important antimicrobials, IA: important antimicrobials; WOAH: World Organisation for Animal Health, VCIA: veterinary critically important antimicrobials, VHIA: veterinary highly important antimicrobials; EMA: European Medicine Agency, B: restrict, C: caution, D: prudence; 13-1 to 20-2: 2013-1 to 2020-2.
